# How morphology affects optical trapping of red blood cells in health and disease

**DOI:** 10.1364/BOE.609102

**Published:** 2026-09-30

**Authors:** Emir Erdem, Gökberk Kabacaoğlu, Giovanni Volpe, Agnese Callegari, Luca Biancofiore

**Affiliations:** 1 Department of Mechanical Engineering, Bilkent University, Ankara, Turkey; 2 Department of Computer Science, Durham University, Durham, United Kingdom; 3 Department of Physics, University of Gothenburg, Gothenburg, Sweden; 4 Department of Industrial and Information Engineering and Economics, University of L’Aquila, L’Aquila, Italy

## Abstract

Optical tweezers are widely used to manipulate microscopic particles and biological cells through optical forces and torques, yet how cell shape controls trapping response has not been fully elucidated. Here, we develop a computational framework to investigate single-beam optical trapping of red blood cells with physiologically and pathologically relevant deformed morphologies. Representative cell shapes are generated parametrically and analyzed using static displacement and rotation tests, together with Brownian dynamics simulations. We show that all considered morphologies remain laterally and orientationally confined in a single-beam trap, while axial localization is weaker by pure optical means and is experimentally realized by the balance between the optical force and the buoyancy-corrected cell weight. Cell geometry strongly affects translational and rotational stiffness, preferred orientation, and Brownian confinement. In particular, shape-induced anisotropy produces distinct dynamical signatures, indicating that optical trapping measurements may be sensitive to morphological alterations beyond simple changes in cell size or volume. These results establish a quantitative connection between red-blood-cell morphology, trap stiffness, and dynamics, providing a computational framework for interpreting morphology-dependent optical trapping experiments.

## 1. Introduction

Red blood cells (RBCs), or erythrocytes, are highly deformable cells responsible for oxygen transport in vertebrates. In humans, healthy RBCs exhibit a characteristic biconcave disk shape, which provides a high surface-area-to-volume ratio and enables efficient gas exchange while preserving the mechanical flexibility required to traverse narrow microcapillaries [1,2]. Changes in RBC morphology are associated with a wide range of physiological and pathological conditions, making cell shape an important indicator of altered biomechanical behavior [1]. Under hydrodynamic stresses, confinement, aggregation, or pathological membrane alterations, RBCs can undergo substantial shape transformations, including elongation, spiculation, constriction, and doublet formation [3,4].

Optical trapping exploits the transfer of momentum from light to matter: a tightly focused laser beam exerts forces and torques on dielectric particles through refraction and reflection of light rays [5,6]. Since its first demonstration by Arthur Ashkin [5], optical tweezers have become a standard tool for the non-contact manipulation of microscopic objects, including biological cells [7,8]. Recent work has further combined holographic optical tweezers with optical-sectioning microscopy for three-dimensional imaging and manipulation of suspended cells [9]. In particular, optical trapping has been extensively applied to RBCs for the investigation of cell mechanics, deformability, membrane fluctuations, and morphology-dependent behavior [10,11]. Optical tweezers have also been used to characterize altered mechanical responses in diseased and mechanically modified RBCs, highlighting the relationship between cell deformability, morphology, and optical manipulation [12]. A healthy RBC can be stably trapped using a single-beam optical tweezer, where its biconcave geometry generates restoring optical torques that align the cell in a folded configuration while remaining free to rotate about the optical axis [10,11]. However, previous experimental and numerical studies have primarily focused on healthy RBCs, pathologically altered cells under specific conditions, or particular trapping configurations, while systematic investigations of broader classes of physiologically and pathologically relevant RBC morphologies remain comparatively limited.

To address this gap, we consider a set of simplified deformed RBC geometries designed to approximate physiologically and pathologically relevant deformations, including dumbbell, four-bumped, eight-bumped, necked, and oblate shapes (see Fig. 1). The dumbbell geometry mimics RBC doublets (rouleaux formation) driven by plasma protein-mediated aggregation [13,14]. The four- and eight-bumped geometries approximate echinocyte-like cells with membrane protrusions arising from metabolic depletion, adenosine triphosphate (ATP) depletion, calcium imbalance, or bilayer-couple effects [15–18]. The necked geometry represents strong mechanical constriction, as experienced during passage through splenic inter-endothelial slits and narrow microcapillaries [3,19,20]. Finally, the oblate geometry approximates anisotropically deformed RBC morphologies, including transient deformation under shear flow and elongated pathological phenotypes such as hereditary elliptocytosis [3,21]. Such morphology transitions and deformation-dependent dynamics are known to arise in confined microflows and strongly influence RBC transport, migration, and hydrodynamic behavior in microfluidic environments [4,22].

**Fig. 1.**
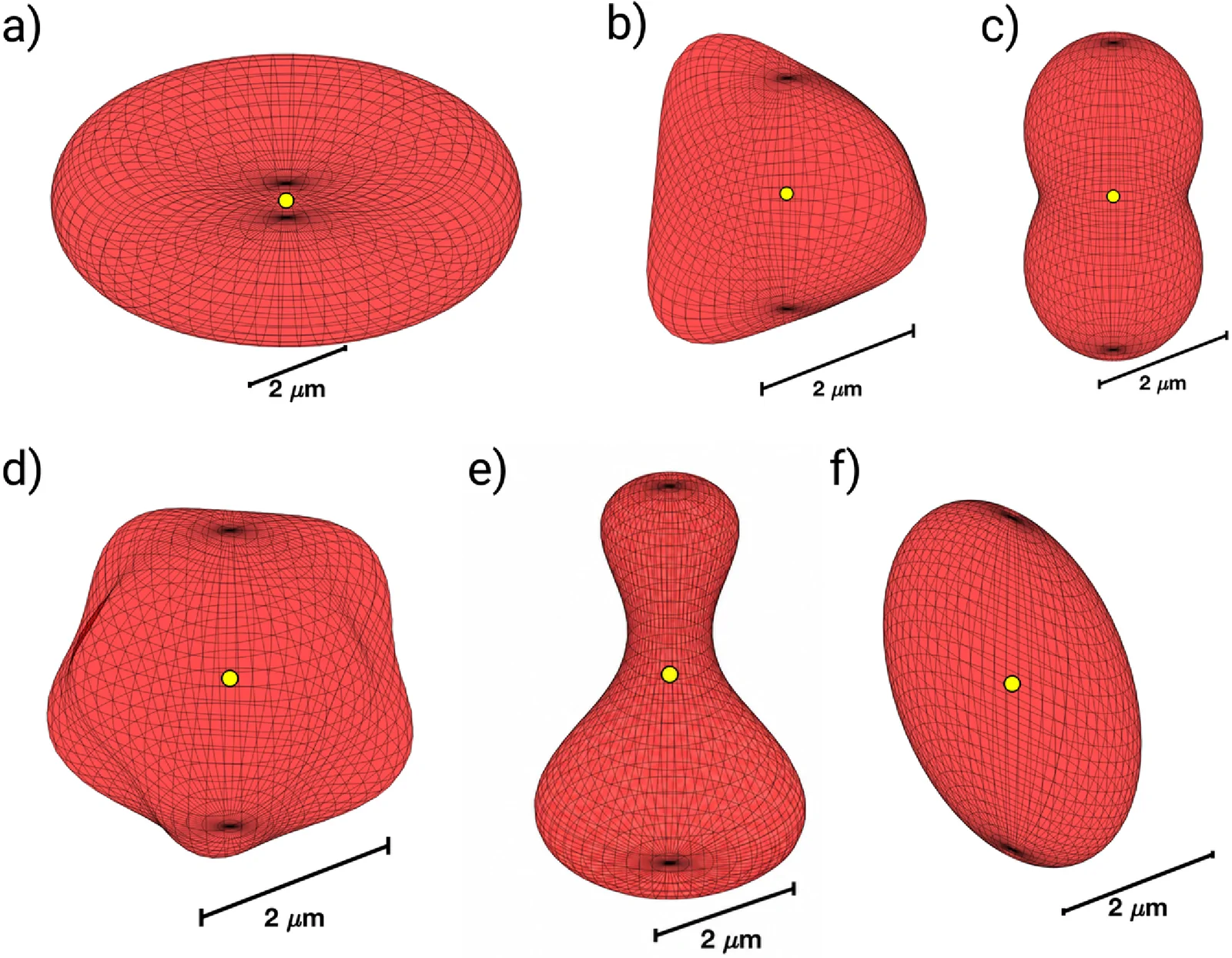
**RBC morphologies used in this study.** (a) uniform, healthy RBC, (b) four-bumped cell, (c) dumbbell, (d) eight-bumped cell, (e) neck and (f) oblate. For visual comparison, all morphologies are plotted at equal enclosed volume; scale bars are 2μm . Natural (unscaled) bounding-box dimensions (width × depth × length) used in the simulations are: (a) 7.8×7.8×2.0μm ; (b) 2.9×2.9×2.4μm ; (c) 1.6×1.6×3.2μm ; (d) 2.2×2.2×2.0μm ; (e) 1.2×1.2×2.0μm ; (f) 0.7×2.0×2.0μm . The volumes used in the optical-trapping and Brownian-dynamics simulations are given in Table 1.

**Table 1.** Geometric properties of the cell morphologies: volume (*μ*m^3^) and surface area (*μ*m^2^)

	Healthy (biconcave)	Dumbbell	Eight-bumped	Neck	Four-bumped	Oblate
Volume	92.9	5.236	4.271	0.922	8.273	1.531
Surface area	133.7	16.200	13.505	5.696	21.164	7.783

The aim of this work is to systematically investigate how these morphology-induced geometric variations influence optical trapping. In contrast to previous studies that primarily focused on equilibrium trapping configurations or healthy RBC geometries, we combine static force and torque analysis with dynamic Brownian simulations to establish a quantitative link between cell geometry, trap stiffness anisotropy, and dynamical confinement. In particular, the dynamic analysis enables characterization of morphology-dependent fluctuations, confinement distributions, mean-squared displacements, and orientational relaxation behavior, providing insight into how trapping signatures vary across different RBC morphologies. By integrating morphology generation, geometrical-optics trapping calculations, and anisotropic hydrodynamic diffusion within a single framework, this work provides a unified comparison of multiple deformed RBC geometries under optical confinement.

The remainder of this paper is organized as follows. Section 2 introduces the mathematical description of the healthy and deformed RBC geometries. Section 3 describes the optical trapping configuration, computational framework, and validation procedure. Section 4 presents the static analysis of trapping forces, torques, and stiffness properties for the different morphologies. Section 5 investigates the corresponding Brownian dynamics and dynamical confinement behavior. Finally, conclusions and perspectives for morphology-dependent optical trapping studies are discussed in Section 6.

## 2. Mathematical model

The biconcave shape of a healthy RBC is described using the classical Evans–Fung model [2], which captures the characteristic concave thickness profile of the cell (see Fig. 1(a)). In this parametrization, the geometry is controlled by a small set of parameters that determine the overall size, thickness distribution, and degree of concavity. In this work, the model is implemented in the following [23], where the shape coefficients are expressed in terms of physically meaningful geometric quantities such as the cell radius and minimum and maximum thickness. The corresponding parameter values used in the simulations are given in the Supplement 1 Section S1.

Deformed morphology surfaces are represented as a smooth parametric map on the sphere,

(1) $$X(\theta ,\phi )=(a_{x}\;\rho (\theta ,\phi )\;\text{sin}\;\theta \;\text{cos}\;\phi ,\;a_{y}\;\rho (\theta ,\phi )\;\text{sin}\;\theta \;\text{sin}\;\phi ,\;a_{z}\;\rho (\theta ,\phi )\text{cos}\;\theta ),$$

where θ∈[0,π] is the polar angle and ϕ∈[0,2π) the azimuthal angle on a Gauss–Legendre × uniform grid. The factors $a_{x},a_{y},a_{z}>0$ apply global anisotropic scaling, and the scalar field ρ(θ,ϕ)>0 modulates the radius to encode local deformations.

1. **Four-bumped cell**: A smooth, low-mode spiculation with four prominent protrusions (see Fig. 1(b)):
(2) $$\rho (\theta ,\phi )=1+0.2\;\text{exp}\;(-3\;\text{Re}\{Y_{3}^{2}(\theta ,\phi )\}),$$
  where $Y_{3}^{2}$ is the degree 3, order 2 spherical harmonic. The constants 0.2 and 3 control bump amplitude and localization, respectively.
2. **Dumbbell**: A two-lobed geometry mimicking RBC doublets/rouleaux (see Fig. 1(c)):
(3) $$\rho (\theta ,\phi )=1+\text{Re}\left\{Y_{2}^{0}(\theta ,\phi )\right\},$$
  where $Y_{2}^{0}$ is the (complex) spherical harmonic of degree 2 and order 0, and Re{⋅} takes its real part. Here θ,ϕ are the polar and azimuthal angles, respectively.
3. **Eight-bumped cell**: An azimuthal mode–4 modulation that produces eight protrusions (see Fig. 1(d)):
(4) $$\rho (\theta ,\phi )=\text{exp}\;(0.5\;{\text{sin}}^{4}\theta \;\text{cos}\;\theta \;\text{cos}\;(4\phi )).$$
  Here the factor ${\text{sin}}^{4}\theta \;\text{cos}\;\theta$ gates the modulation toward mid-latitudes and sets fore-aft asymmetry, while cos⁡(4ϕ) imposes eight bumps around the azimuth. The coefficient 0.5 sets overall bump strength.
4. **Neck**: A waist-like constriction representing strong mechanical squeezing. Let z=cos⁡θ and define a constriction factor (see Fig. 1(e))
(5) $$s(z)=1-c_{f}\;\text{cos}\;\;\left(\frac{\pi }{2}(z-0.2)\right),$$
  with $c_{f}\in (0,1)$ controlling neck severity (default $c_{f}=0.8$ ). The local in-plane radius is
(6) $$r(\theta )=\sqrt{1-z^{2}}\;s(z),$$
  and the surface is given explicitly by
(7) X(θ,ϕ)=(r(θ)cos⁡ϕ,r(θ)sin⁡ϕ,z).
  Here *θ* and *ϕ* retain their spherical meanings; z=cos⁡θ , and *c_f_* is a user-chosen scalar that narrows or widens the neck.
5. **Oblate**: A globally flattened (elliptical) cell obtained by axial compression (see Fig. 1(f)):
(8) $$\rho (\theta ,\phi )=1,\;(a_{x},a_{y},a_{z})=(0.366,\;1,\;1).$$
  Thus
(9) X(θ,ϕ)=(0.366sin⁡θcos⁡ϕ,sin⁡θsin⁡ϕ,cos⁡θ).
  Here $a_{x}=0.366$ sets the target oblateness while $a_{y}=a_{z}=1$ .

Table 1 shows the volume and the surface area of each morphology.

The healthy biconcave geometry, based on the Evans–Fung parametrization ( Supplement 1, Section S1), has a diameter of 2r=7.82μm and the thickness parameters are consistent with experimental erythrocyte geometry [1,2]. Because this study focuses on how morphology affects optical trapping, the deformed cells are simplified parametric surfaces representing well-documented physiological and pathological shape classes, rather than fits to individual-cell imaging data. Explicitly, the four- and eight-bumped morphologies are constructed with four and eight protrusions and bump heights of 0.51μm and 0.18μm , respectively, as reduced echinocyte-like surfaces; fully developed echinocytes (echinocyte III) typically display on the order of tens of spicules (about 25–50), with characteristic spicule heights of roughly 0.6−1μm [15,16]. The dumbbell and oblate geometries are strongly anisotropic, with length-to-width and long-to-short aspect ratios of approximately 2.0 and 2.7, respectively, consistent with doublet/rouleaux-like aggregation and elongated or elliptocytic phenotypes [3,13,14,21]. The neck morphology is a strongly constricted two-lobed shape (waist-to-maximum diameter ratio ≈ 0.31 for $c_{f}=0.8$ ), representing the geometric class of mechanically squeezed cells associated with passage through narrow capillaries and splenic interendothelial slits [3,19,20].

Standard differential–geometric operators on the surface of these geometries (surface normals, metric coefficients, mean and Gaussian curvatures, as well as surface gradient, divergence, and Laplace–Beltrami operators) are used to evaluate ray–surface interactions, and hence optical forces and torques. Further details on the numerical construction of the cell geometries and the associated geometric operators, as well as their use in the computation of optical forces and torques, are provided in Supplement 1 (Section S5).

## 3. Computational and optical setup

This section describes the computational framework used to evaluate optical forces and torques on the different red blood cell morphologies. We first introduce the ray-optics model, optical parameters, and numerical implementation used in the OTGO simulations (Section 3.1). We then describe the specific setups adopted for the static displacement/rotation analysis (Section 3.2) and for the Brownian dynamics simulations (Section 3.3). Finally, the framework is validated against the known trapping behaviour of a healthy biconcave RBC (Section 3.4).

### 3.1. Optical model and numerical implementation

Computations in this study were performed using OTGO (Optical Tweezers in the Geometrical Optics approximation), version 1.0.0 [24]. The ray-optics approximation was adopted because the characteristic length-scales of the modeled RBC geometries are larger than the trapping wavelength, placing the system in the geometrical-optics regime [25]. In this framework, the incident laser beam is represented as a collection of discrete rays, and optical forces and torques are computed from momentum transfer based on geometrical optics.

Optical trapping was modeled using a Gaussian beam with a power of 5mW , focused through a high–numerical-aperture objective with NA=1.3 . A tightly focused single-beam configuration was considered, with beam propagation along the positive *z*-axis. The origin of the laboratory coordinate system was placed at the beam focus, which served as a fixed reference point. The refractive indices of the RBC interior and surrounding aqueous medium were taken as $n_{\text{RBC}}=1.38$ and $n_{\text{medium}}=1.33$ , respectively, consistent with previous ray-optics modeling of erythrocytes [26].

In the geometrical-optics (GO) calculation, the vacuum wavelength does not enter the ray-tracing algorithm used to compute forces and torques; the optical response depends on the refractive indices of the cell and the medium. The validity of the GO approximation, however, depends on how large the object is compared with the wavelength in the surrounding medium. We take a trapping wavelength of $\lambda_{0}=1064\;\text{nm}$ , which is commonly used in biological optical tweezers [6], so that in water $\left(n_{\text{medium}}=1.33\right)$ the wavelength is $\lambda_{m}=\lambda_{0}/n_{\text{medium}}\approx 0.80\;\mu m$ . For the eight-bumped cell (overall size ∼2.2μm ), the size-to-wavelength ratio is about 2–3, which is reasonable for a rough GO estimate. However, the surface bumps are much smaller (height ∼0.18μm ) and are close to or smaller than *λ_m_*, so the approximation is more borderline for these fine features. The neck cell (width ∼1.2μm ) is similarly close to the GO limit. The convergence tests in Figs. S1–S3 show that the GO solver is numerically stable. These tests check the numerical convergence of the GO solver, not its accuracy relative to full-wave electromagnetics.

Light polarization can strongly affect optical forces and torques and is naturally included in full-wave approaches such as T-matrix and FDTD methods [27,28]. By construction, the standard geometrical-optics formulation used here does not account for polarization-dependent effects. Polarization can be introduced phenomenologically within a ray-optics framework [29], but such treatments are not equivalent to a full-wave calculation: they typically assume that the polarization carried by each ray is deposited onto the object (affecting the torque) at the first relevant scattering event only. In this work we therefore employ the standard non-polarized GO model. Extending the present morphology comparison to polarized illumination with full-wave methods is an important direction for future study.

The numerical parameters of the geometrical-optics solver were selected to balance accuracy and computational efficiency. Convergence tests were performed for the ray discretization, surface tessellation, and number of scattering events, showing that moderate parameter values are sufficient to obtain stable optical forces and torques. The final choices were adopted consistently across all simulated geometries. Further details of these analyses are provided in Figs. S1–S3 of Supplement 1 and in Section S2.

### 3.2. Static analysis setup

In the static analysis, the origin of the coordinate system coincides with the beam focus. The equilibrium trapping position (trap center), however, is set by the balance of optical forces and torques and does not necessarily coincide with either the beam focus or the particle geometric center, as illustrated in Fig. 2.

**Fig. 2.**
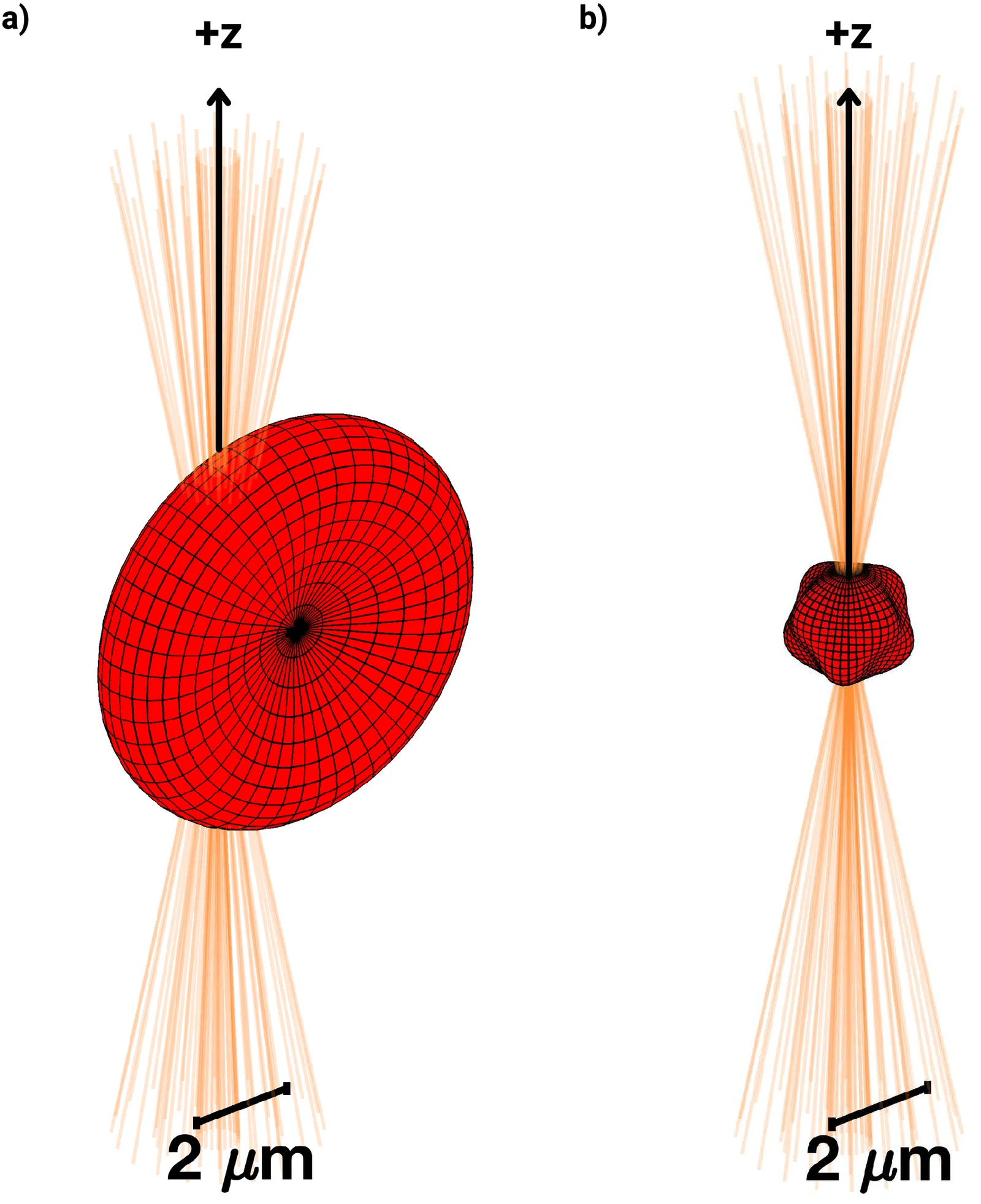
**Representation of the optical trapping of RBCs via a single beam optical tweezers.** (a) Biconcave RBC and (b) eight-bumped cell pictured in their own typical trapping position. The origin of the coordinate system is set at the focus of the beam. As shown in (a), the biconcave RBC is trapped preferentially through its thick rim, and its geometrical center lies off-axis, in agreement with the literature [11]. On the other hand, the eight-bumped cell is trapped on-axis. The orange lines represent the constituent rays of the focused beam. For clarity, only the more central rays are shown with a down-scaled discretization, while the more peripheral ones are omitted. We assume a single beam optical tweezers that propagates in the positive z direction. In (b), the eight-bumped cell is trapped on-axis. Both panels share the same length scale (scale bars, 2 *μ*m).

The cells were initially placed near the beam focus (origin of the laboratory frame), which serves as a reference point for the displacement analysis. Dumbbell, four-bumped, eight-bumped, and oblate cells were initialized with their geometric centers at the origin. In contrast, the neck cell was positioned (0.6μm) above the origin such that its lower spherical lobe was approximately aligned with the beam focus. This adjustment accounts for the asymmetric geometry of the neck morphology and ensures that the region most strongly interacting with the optical field is centered. This choice does not affect the generality of the results, as the trapping position is determined by the force balance and the analysis is based on relative displacements from the equilibrium configuration.

All rotational force and torque sweeps start from a fixed reference orientation in which the cell body axes coincide with the laboratory axes. For the healthy biconcave RBC and the oblate cell this places the disk in a vertical (edge-on) configuration relative to the beam axis; for elongated shapes (dumbbell, neck) the symmetry axis is aligned with the optical axis. Rotational sweeps then rotate the cell about one laboratory axis at a time from this pose. Translational force–displacement sweeps use the same reference orientation for the healthy, dumbbell, neck, and oblate cells. For the healthy biconcave RBC, Fig. 3(b)–(d) are computed in this edge-on reference orientation (body axes aligned with the laboratory axes). Translational stiffnesses in Table 2 are obtained from linear fits at the off-center lateral equilibria on those force curves (rim trapping), not at the dynamically tilted pose shown in (3)(a). For the four- and eight-bumped cells, which exhibit nonzero restoring torques away from this pose, translational sweeps (including axial *z*-displacements) are instead performed at the stable orientation identified from the torque–rotation curves ( $\theta_{x}\approx 0.97$ and 1.29 rad, respectively), so that the reported stiffnesses correspond to a rotationally preferred trapping configuration.

**Fig. 3.**
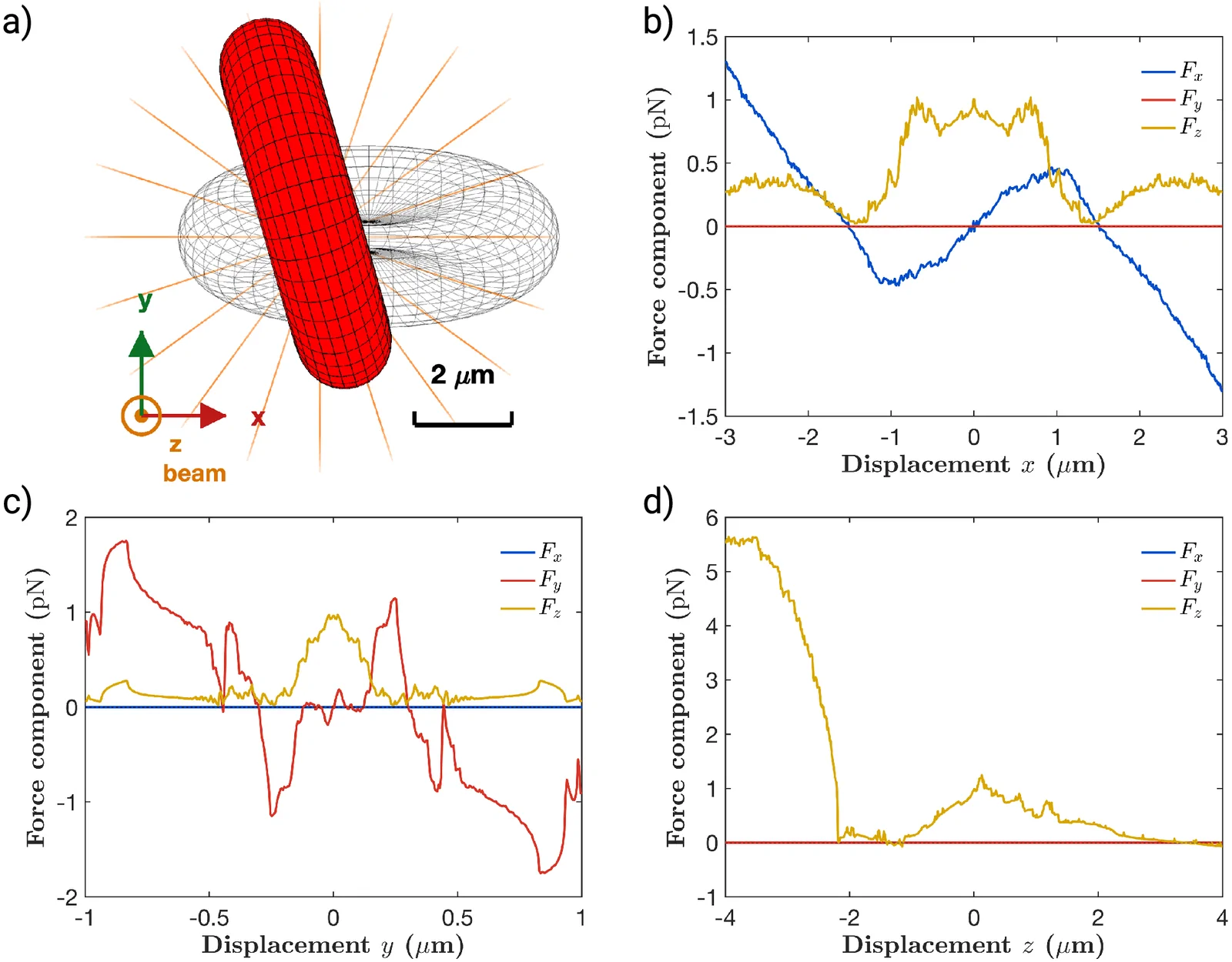
Trapping of a healthy RBC. (a) Initial (wireframe) and final (solid) configurations after 100s of Brownian dynamics. The cell starts 1μm off-axis along *x* and rotated by 0.4π radians $\left(\approx 72^{\circ }\right)$ about the *x*-axis. Optical forces and torques drive it slightly off-center and into a tilted trapped pose (reached after ∼15s ). Laboratory axes are shown; the beam propagates along +z (out of the page). For this near-axisymmetric geometry the single-beam trap confines the cell laterally and in tilt, but does not restore rotation about the beam axis (nor about the cell’s symmetry axis), so the final solid pose in (a) need not coincide with the static analysis orientation. (b)–(d) Optical force components for static displacements along *x*, *y*, and *z*, respectively, in the edge-on reference orientation of Sec. 3.2 (body axes coinciding with the laboratory axes). Translational stiffnesses in Table 2 are evaluated at the off-center lateral equilibria on these curves.

**Table 2.** Translational trap stiffness values for different cell geometries (pN/μm)

Axis	Healthy (biconcave)	Dumbbell	Eight-bumped	Neck	Four-bumped	Oblate
*x*-axis	0.931	1.913	1.891	2.737	1.240	5.737
*y*-axis	3.668	1.913	1.373	2.737	1.241	1.301

### 3.3. Dynamic analysis setup

The motion of a cell immersed in a liquid medium and confined within an optical trap is governed by the interplay between thermal fluctuations, viscous hydrodynamic drag from the surrounding medium, and optical forces exerted by the trapping field [6,30]. The translational motion of a spherical particle can be described by a single diffusion coefficient. However, most biological cells are non-spherical and anisotropic, leading to more complex hydrodynamic behavior. In such cases, the dynamics are described by a generalized diffusion tensor D rather than a scalar diffusivity. The diffusion tensor was computed using the software winHYDRO++ [31,32], which implements bead-model hydrodynamics to evaluate translational, rotational, and coupling diffusion coefficients. The calculations assume an unbounded (bulk) fluid environment, neglecting hydrodynamic interactions with nearby boundaries, which are known to modify translational and rotational drag near surfaces [33]. Further details of this approach are provided in Supplement 1 (Section S4.1).

The dynamic behavior of each trapped cell was simulated using overdamped Brownian dynamics [34], where thermal fluctuations, hydrodynamic resistance, and restoring optical forces jointly determine the motion. At each time step, stochastic displacements and rotations were computed consistently with the fluctuation–dissipation theorem, allowing the coupled translational and rotational dynamics of the cell to be resolved. Further implementation details are provided in Supplement 1 (Section S4.2).

### 3.4. Validation

To validate the computational framework, the dynamic behavior of a healthy RBC in a single-beam optical tweezer was simulated, including Brownian motion. Thermal fluctuations were modeled with a Gaussian white-noise term, and the particle dynamics were integrated over 100 s with Δt=0.01s . This time step is smaller than the trap relaxation time $\tau_{\text{OT}}=\gamma /k\approx 1.5\times 10^{-2}\;s$ estimated for the healthy RBC from its diffusion tensor and lateral trap stiffness (Fig. 3), and much larger than the momentum relaxation time $\left(\tau_{m}∼10^{-4}\;s\right)$ [35], ensuring stable integration of the overdamped dynamics.

The healthy RBC is stably trapped in a tilted configuration, with its major axis approximately aligned with the beam propagation direction (+z) , as shown in Fig. 3(a) and Fig. 2(a), consistent with previous ray-optics simulations [35]. For this near-axisymmetric biconcave geometry, the single-beam trap confines the cell laterally and in tilt, but does not generate a restoring torque about the optical (*z*) axis; the cell remains free to spin about the beam.

To further characterize the trapping response, the optical force components were recorded as the cell was displaced along each of the three Cartesian axes. The results of both the dynamic Brownian simulations and the static force analysis are shown in Fig. 3.

## 4. Static analysis

The static analysis is used to characterize how each red blood cell morphology responds to controlled displacements and rotations within the optical trap. We first exploit the symmetry of the different geometries to identify the independent force and torque responses that need to be computed (Section 4.1). We then use the near-equilibrium linear regime to extract translational and rotational trap stiffnesses from the force–displacement and torque–rotation curves (Section 4.2). Finally, we relate the resulting translational stiffnesses to simple geometric descriptors of the cell shapes, in order to assess how morphology controls trapping strength (Section 4.3).

### 4.1. Symmetry-based force and torque response

The geometries can be grouped according to their symmetry with respect to the optical (*z*) axis. For shapes that are rotationally symmetric, namely the dumbbell and neck morphologies (see Fig. 1(c) and (e) respectively), the rotational symmetry axis is aligned with the beam propagation (*z*) axis. In these cases, displacements along the *x*- and *y*-axes produce nearly identical force and torque responses, and it is therefore sufficient to consider a single transverse direction. Figure 4 shows representative force and torque responses for the axial-symmetric cases.

**Fig. 4.**
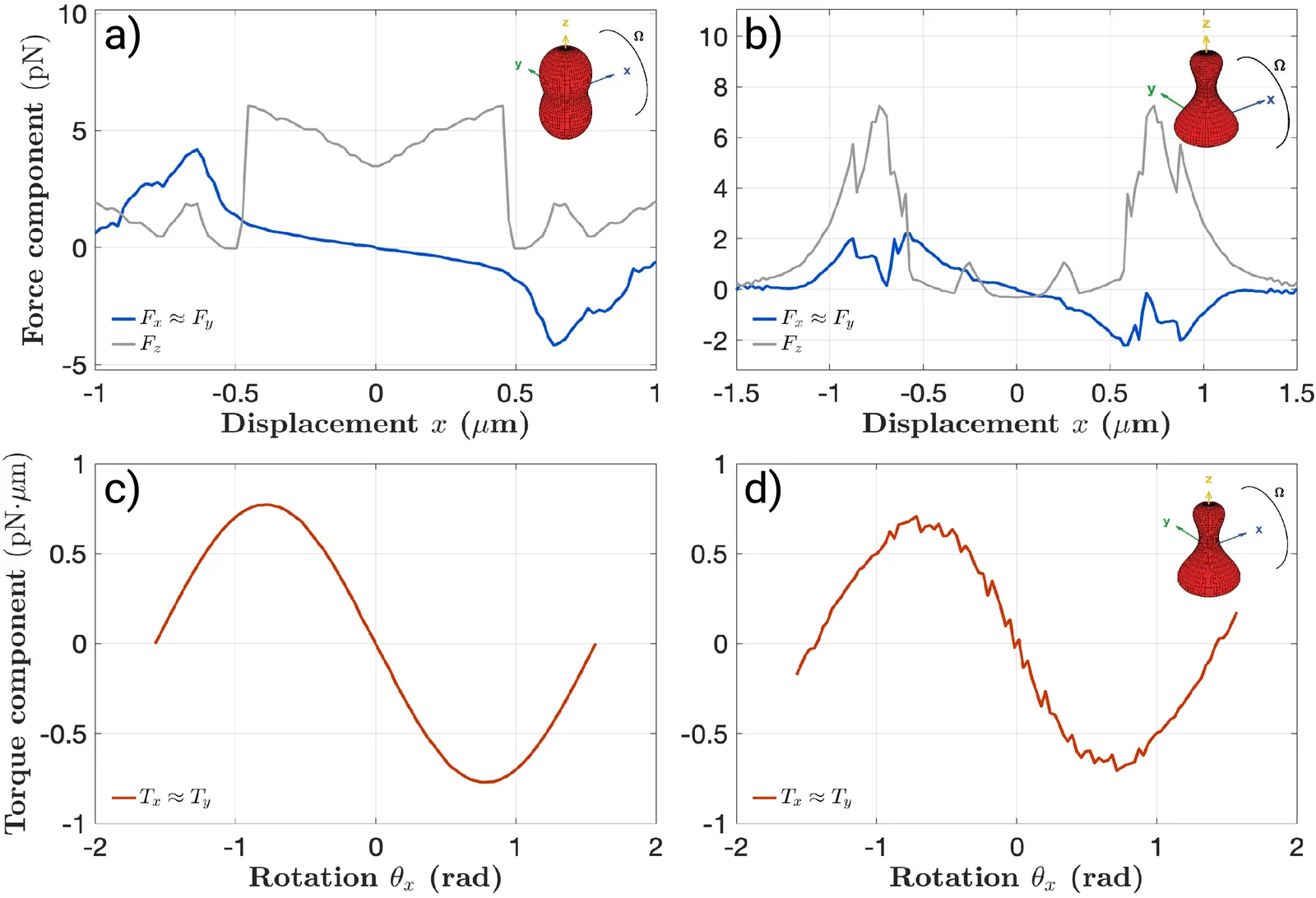
**Force and torque responses of rotationally symmetric red blood cell morphologies under optical trapping.** Top row: optical forces for *x*-displacements of the (a) dumbbell and (b) neck cells (± 1–1.5 *μ*m from the trap center). Blue: lateral force $\left(F_{x}\approx F_{y}\right)$ ; gray: axial force $\left(F_{z}\right)$ . Bottom row: corresponding torques versus rotation angle *θ_x_* for the (c) dumbbell and (d) neck (orange; $T_{x}\approx T_{y}$ ). Both morphologies use the reference orientation $\theta_{x}=0$ for the displacement sweeps. Forces are in piconewtons (pN) and torques in piconewton–micrometers (pN⋅μm) . Approximate sizes (width×depth×length): dumbbell ∼1.6×1.6×3.2μm ; neck ∼1.2×1.2×2.0μm . Among these shapes, the neck is closest to the geometrical-optics limit relative to $\lambda_{m}\approx 0.80\;\mu m$ (Sec. 3.1).

In contrast, the four-bumped, eight-bumped and oblate cells (see Fig. 1(b), (d) and (f) respectively) lack full rotational symmetry about the *z*-axis and must be analyzed separately along the *x*- and *y*-directions. For the oblate geometry, rotational symmetry about the *x*-axis eliminates the need to consider rotations about that axis. Figure 5 shows the corresponding responses for the non-symmetric geometries, where directional dependence becomes apparent. For all geometries, transverse displacements and rotations generate restoring forces and torques, indicating stable trapping configurations.

**Fig. 5.**
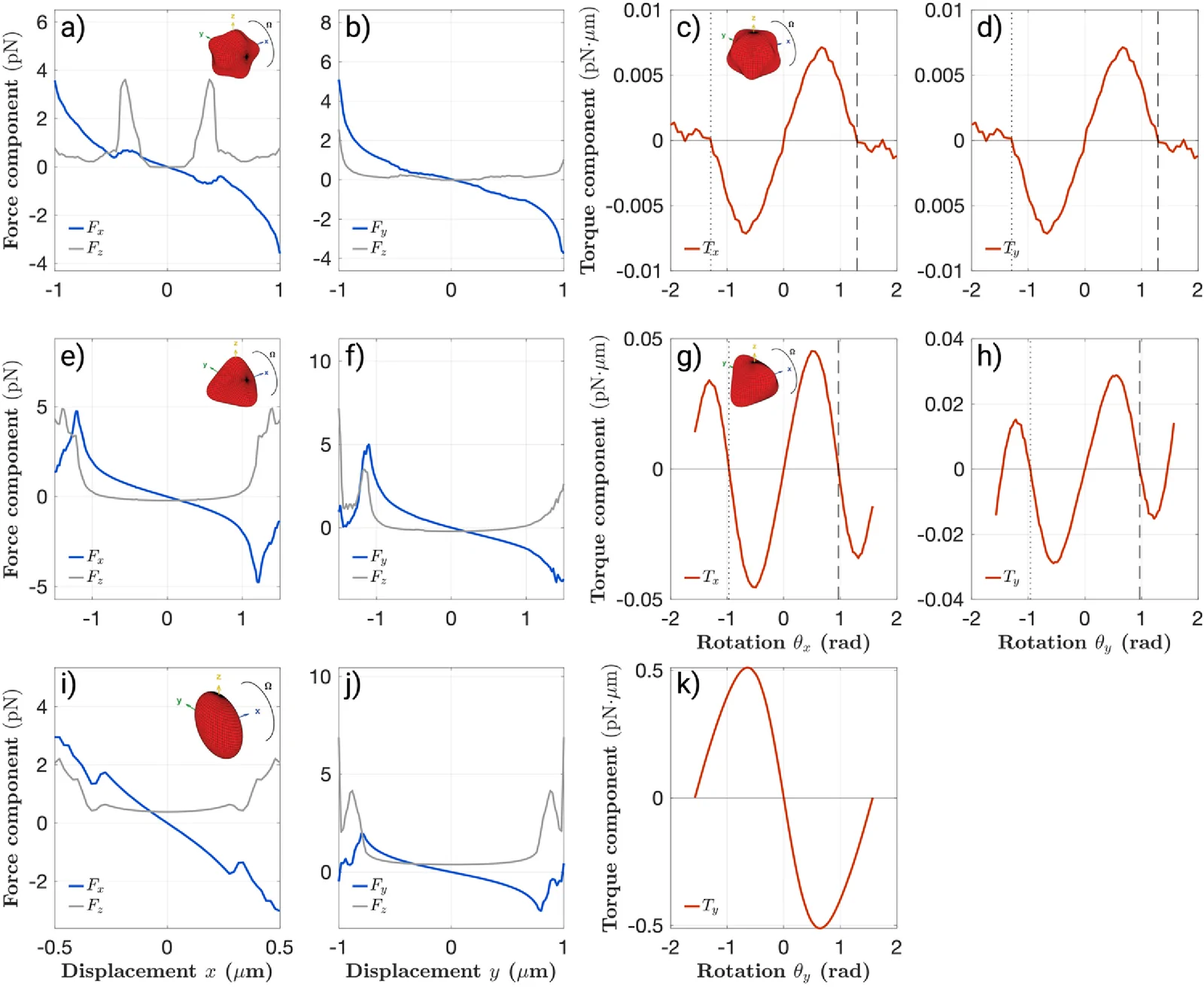
**Force and torque responses for morphologies that lack full rotational symmetry about the optical (*z*) axis.** Top row: eight-bumped cell under (a) *x*-displacement, (b) *y*-displacement, (c) rotation about *x*, and (d) rotation about *y*. Middle row: four-bumped cell under (e) *x*-displacement, (f) *y*-displacement, (g) rotation about *x*, and (h) rotation about *y*. Bottom row: oblate cell under (i) *x*-displacement, (j) *y*-displacement, and (k) rotation about *y* (no *x*-rotation shown due to axial symmetry). In (c)–(d) and (g)–(h), dashed (dotted) vertical lines mark the stable tilt equilibria θ=±1.29rad (eight-bumped) and θ=±0.97rad (four-bumped). For the eight-bumped cell, panels (c) and (d) show the same T(θ) curve by four-fold symmetry about *z* $\left(T_{x}(\theta_{x})\equiv T_{y}(\theta_{y})\right)$ . Displacement sweeps use the stable tilt for the bumped cells ( $\theta_{x}\approx 1.29\;\text{rad}$ for eight-bumped; $\theta_{x}\approx 0.97\;\text{rad}$ for four-bumped) and the reference orientation for the oblate cell. For displacements, lateral components (*F_x_* or *F_y_*, blue) and axial components (*F_z_*, gray) are reported in piconewtons (pN); for rotations, restoring torques (*T_x_* or *T_y_*, orange) are in piconewton–micrometers (pN⋅μm) . Approximate sizes (width×depth×length): eight-bumped ∼2.2×2.2×2.0μm ; four-bumped ∼2.9×2.9×2.4μm ; oblate ∼0.7×2.0×2.0μm .

### 4.2. Linear trap stiffness approximation

Near the trap center, the optical forces and torques exhibit an approximately linear dependence on displacement and rotation, such that F≈−kx and τ≈−κθ , where *k* and *κ* denote the translational and rotational trap stiffness, respectively [6]. The trap stiffness can therefore be estimated by performing a linear fit to the force–displacement and torque–rotation curves in the vicinity of the equilibrium position and extracting the corresponding slopes. Tables 2 and 3 summarize the translational and rotational stiffness values for all cell geometries along the *x*- and *y*-axes. The nearly spherical geometry of the eight-bumped cell gives rise to only weak rotational torques ( $\kappa_{x}=\kappa_{y}\approx 0.008\;\text{pN}\cdot \mu m/\text{rad}$ at the stable tilt $\theta^{\text{eq}}\approx 1.29\;\text{rad}$ ; Table 3). By four-fold symmetry about the optical axis, the $T_{x}(\theta_{x})$ and $T_{y}(\theta_{y})$ responses coincide, so both rotational stiffnesses are reported. Nevertheless, restoring forces along *x* and *y* still confine the cell; at the preferred tilt the lateral stiffness remains anisotropic ( $k_{x}=1.891\;\text{pN}/\mu m,k_{y}=1.373\;\text{pN}/\mu m$ ; Table 2).

**Table 3.** Rotational trap stiffness values for different cell geometries (pN⋅μm/rad)

Axis	Healthy (biconcave)	Dumbbell	Eight-bumped	Neck	Four-bumped	Oblate
*x*-axis	5.050	1.385	0.008	1.593	0.146	symm.
*y*-axis	symm.	1.385	0.008	1.593	0.096	1.229

For completeness, Tables 2 and 3 also include the healthy biconcave RBC. Its force–displacement curves (Fig. 3(b)–(d)) are evaluated in the edge-on reference orientation of Sec. 3.2 (body axes aligned with the laboratory axes). Translational stiffnesses in Table 2 are obtained from linear fits at the off-center lateral force equilibria on those curves, consistent with preferential trapping through the thick rim. Rotational stiffness about *x* $\left(\kappa_{x}=5.050\;\text{pN}\cdot \mu \text{m/rad}\right)$ is obtained from the near-zero linear regime of the torque–rotation response ( Supplement 1, Fig. S5); rotational stiffness about the body *y* symmetry axis is omitted (symm.) because $T_{y}\approx 0$ for that rotation in the analysis configuration. Tables 2 and 3 use the same orientation convention: translational and rotational stiffnesses for a given morphology are evaluated in the same trapping pose (reference orientation, or the stable tilt $\theta^{\text{eq}}$ when that pose differs from the reference).

Finally, axial displacements along the *z*-axis were examined to assess scattering contributions (Fig. 3(d) and Fig. S4). In these single-beam geometrical-optics calculations the axial optical force *F_z_* remains small (sub-pN) near the trapping region and does not exhibit a clear, broad linear restoring well about *z* = 0. This is expected: lateral confinement is provided by gradient forces, whereas the axial force is dominated by scattering. In a typical upright experimental geometry, axial equilibrium is obtained by balancing this weak optical *F_z_* against the buoyancy-corrected cell weight $)∼10^{-3}–10^{-2}\;\text{pN}$ for the deformed morphologies; see Supplement 1, Table S1), rather than by a strong optical restoring spring along *z*. We therefore interpret the axial curves as showing that scattering does not destabilise the trap, while stable lateral (and tilt) confinement is the operative trapping criterion in this study.

### 4.3. Geometric dependence of translational trap stiffness

To quantify the influence of cell morphology on trapping strength, we introduce a simple geometric descriptor based on the equivalent ellipsoidal aspect ratio of each shape. For a given cell surface, the principal axes (a,b,c) are obtained from the covariance matrix of the surface coordinates, and the aspect ratio is defined as a/c , where a≥b≥c (see Supplement 1, Section S3.3).

Figure 6 summarizes the GO volume-rescaled mean lateral stiffness ( Supplement 1, Section S3.4), constructed from the values in Table 2, as a function of aspect ratio. A general increase with notable deviations is observed, indicating that more anisotropic shapes generally experience stronger optical confinement after a GO volume-based size rescaling. In this analysis, we focus on translational trap stiffness, as it provides a consistent scalar measure of confinement strength across all morphologies, whereas rotational stiffness can be negligible or symmetry-dependent for certain geometries (e.g., the eight-bumped cell).

**Fig. 6.**
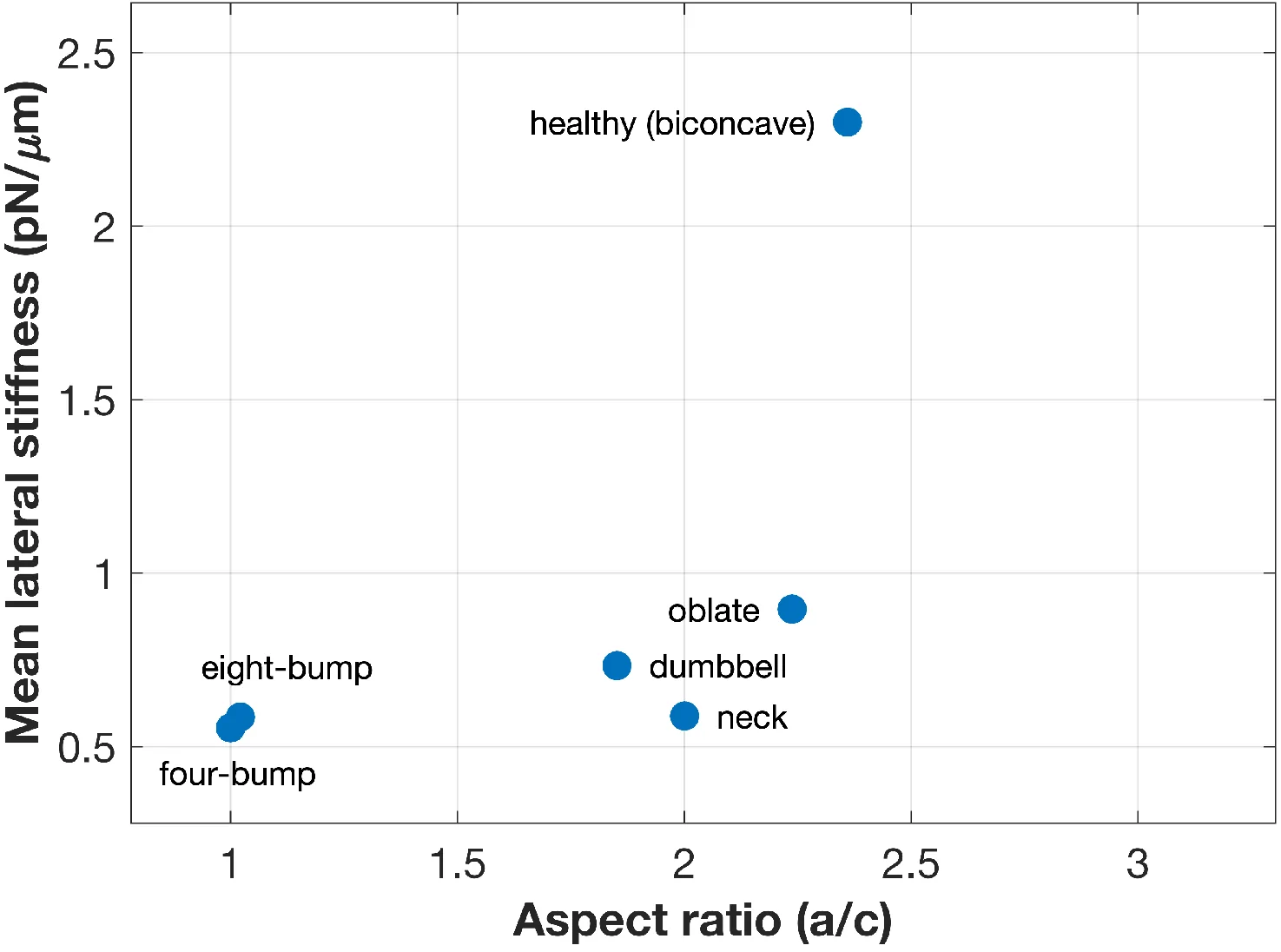
**Volume-rescaled mean lateral trap stiffness as a function of geometric aspect ratio a/c for the different cell morphologies.** All morphologies are compared under the same single-beam optical-tweezers configuration ( $5\;\text{mW},\text{NA}=1.3,n_{\text{RBC}}=1.38,n_{\text{medium}}=1.33$ ; Section 3.1) and the same ray-optics discretization. Each point corresponds to one morphology at its natural enclosed volume and surface area (Table 1), analyzed in its geometry-specific equilibrium trapping configuration, including orientation and trap-center placement (Section 3.2). The plotted quantity is the GO volume-rescaled mean lateral stiffness defined in Supplement 1, Section S3.4, based on $k_{\text{av}}=(k_{x}+k_{y})/2$ from linear fits near equilibrium (Section 4.2, Table 2). The aspect ratio a/c is obtained from the area-weighted equivalent ellipsoid fitted to each surface ( Supplement 1, Section S3.3). The comparison isolates morphology-dependent stiffness at fixed trap parameters after a GO volume-based size rescaling. A general increase in rescaled stiffness with anisotropy is observed, with deviations reflecting local surface features and symmetry. See also Supplement 1, Fig. S6 for the analogous plot versus asphericity.

This behavior can be understood in terms of the effective interaction between the incident beam and the particle geometry. Previous studies on non-spherical particles have shown that optical restoring forces and torques depend sensitively on particle anisotropy, projected area, and refractive deflection of rays [36–38]. Consistent with these observations, morphologies with larger aspect ratios in the present study generally exhibit larger volume-rescaled translational stiffness. In particular, the healthy biconcave cell combines the largest a/c with the largest rescaled stiffness, while the oblate cell also lies toward the high- a/c , high-stiffness end of the set. By contrast, the neck morphology has a comparatively large aspect ratio but a rescaled stiffness close to that of the near-spherical bumped cells, showing that elongation alone does not determine confinement after size correction.

The aspect ratio captures a leading contribution of global geometric anisotropy to trap stiffness. Deviations from a strictly monotonic trend—including the bumped cells near a/c≈1 , the comparatively low rescaled stiffness of the neck morphology at large a/c , and the separation between the dumbbell and oblate cells at intermediate a/c —indicate that local surface curvature, protrusion structure, and symmetry class, which are not encoded in the ellipsoidal aspect ratio alone, also affect ray–surface interactions and therefore trapping stiffness.

Because the morphologies differ substantially in enclosed volume (Table 1), a raw stiffness–aspect-ratio comparison would confound anisotropy with size. Figure 6 therefore uses the GO volume-rescaled stiffness of Supplement 1, Section S3.4, which removes a characteristic-length size contribution while leaving residual morphology-dependent scatter.

Overall, these results demonstrate that geometric anisotropy provides a useful first-order predictor of trap stiffness after size correction, while a more complete geometric descriptor would likely need to incorporate local curvature information alongside the global anisotropy measure.

## 5. Dynamic analysis

The static displacement and rotation analyses provide local measures of the restoring forces and torques generated by the optical trap, and therefore of the translational and rotational stiffness associated with each morphology. To determine whether these restoring effects are sufficient to ensure stable confinement under thermal fluctuations, we performed Brownian dynamics simulations of the different red blood cell geometries. This section first discusses the choice and validation of the stochastic integration time step (Section 5.1), and then examines the resulting Brownian dynamics and morphology-dependent confinement through the center–trap distance, radial probability distributions, mean-squared displacement, and orientational autocorrelation functions (Section 5.2). These quantities allow us to connect the static stiffness measurements to the morphology-dependent translational and rotational confinement observed in the full dynamic simulations.

### 5.1. Simulation time step

The integration time step Δt must be sufficiently small to resolve thermal fluctuations and restoring optical forces, while remaining computationally efficient. In the overdamped regime, the relevant characteristic timescales are given by the trap relaxation time $\tau_{\text{OT}}=\gamma /k$ and the momentum relaxation time $\tau_{m}=m/\gamma$ , where *γ* is the hydrodynamic friction coefficient [6,39].

Using the diffusion tensor obtained for each morphology, friction coefficients were estimated via the fluctuation–dissipation relation $\gamma =k_{B}T/D$ . Combined with the measured trap stiffnesses, this yields translational trap relaxation times in the range $\tau_{t}∼1.5\times 10^{-2}–2\times 10^{-1}\;s$ across all geometries (Table S2); rotational relaxation times $\tau_{r}$ can be substantially longer for weakly torque-confined shapes. A representative momentum relaxation time of $\tau_{m}∼10^{-4}\;s$ was estimated using a typical RBC mass $m∼10^{-11}\;\text{kg}$ [35]. These estimates confirm the overdamped hierarchy $\tau_{\text{OT}}\gg \tau_{m}$ .

Based on these characteristic timescales, the integration time step should satisfy $\tau_{\text{OT}}≳\Delta t\gg \tau_{m}$ . A value of Δt=2ms was selected and validated through numerical tests. As shown in Fig. 7 for a representative dumbbell geometry, stable confinement is maintained for Δt≤3ms , while larger time steps lead to artificial drift and loss of trapping. This confirms that the chosen time step provides a reliable compromise between numerical stability and computational efficiency. Detailed timescale estimates are provided in Supplement 1 (Section S4.2).

**Fig. 7.**
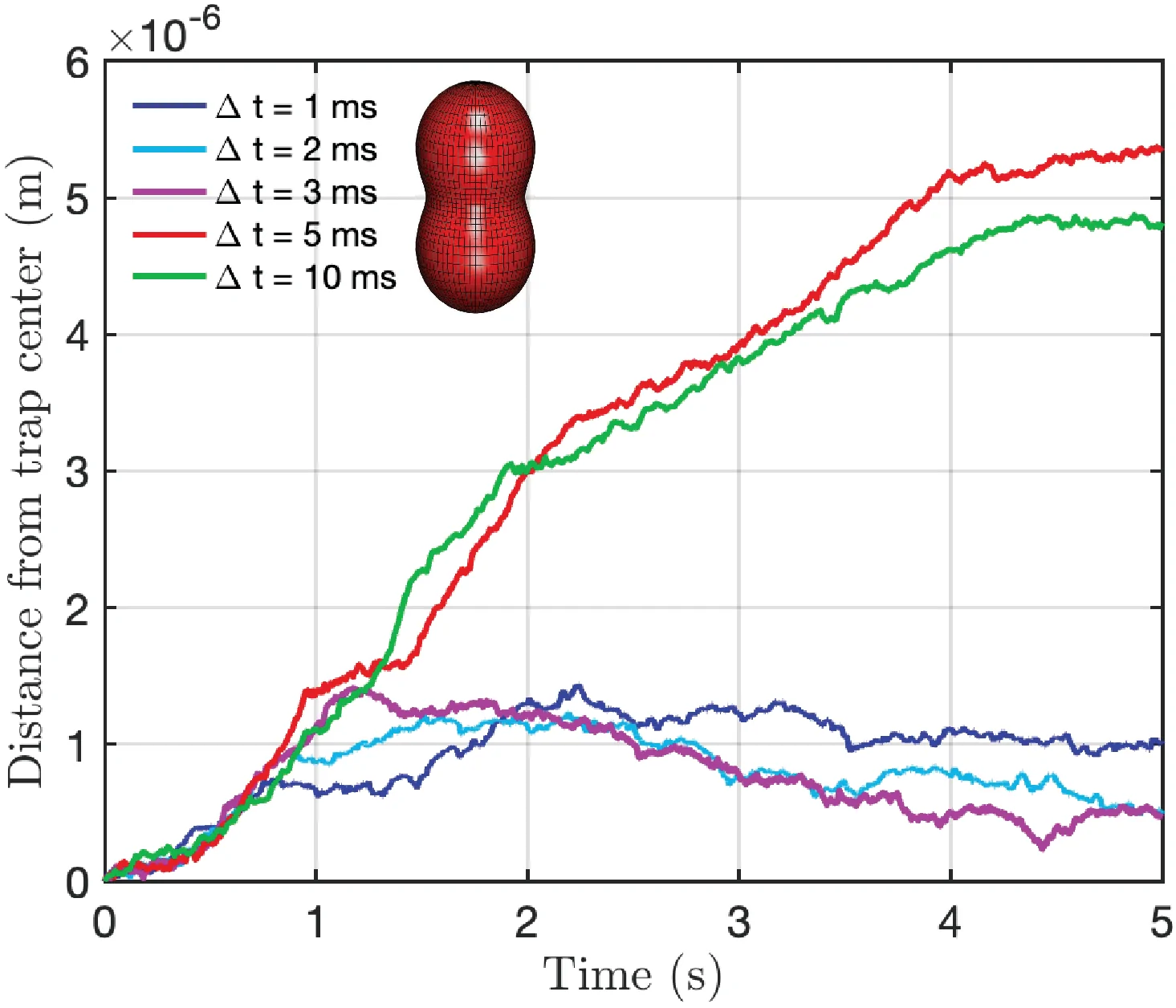
**Time evolution of the dumbbell’s distance from the trap center for different integration time steps Δt .** The plotted distance is the three-dimensional Euclidean distance between the cell center and the beam focus, $r=\sqrt{x^{2}+y^{2}+z^{2}}$ , not a single-axis projection. For Δt≤3ms the cell stays confined (bounded fluctuations in *r*). For Δt=5 and 10ms the integrator is unstable and *r* grows without bound. This supports Δt=2ms as a stable choice for the Brownian dynamics runs.

### 5.2. Brownian dynamics and morphology-dependent confinement

Dynamical trappability was assessed by performing three independent Brownian dynamics simulations for each cell morphology, all initialized near the beam focus with random orientations. The resulting trajectories are summarized in Fig. 8. The time evolution of the center–focus distance $r=\sqrt{x^{2}+y^{2}+z^{2}}$ (full three-dimensional Euclidean distance; (Fig. 8(a–e)) demonstrates that all investigated morphologies remain confined within a bounded region over the full 10 s simulation window. After an initial transient phase, each trajectory fluctuates around a steady mean distance from the focal point, with amplitudes comparable to or smaller than the characteristic cell size. No systematic drift away from the equilibrium trapping region is observed, confirming stable dynamical confinement under thermal fluctuations.

**Fig. 8.**
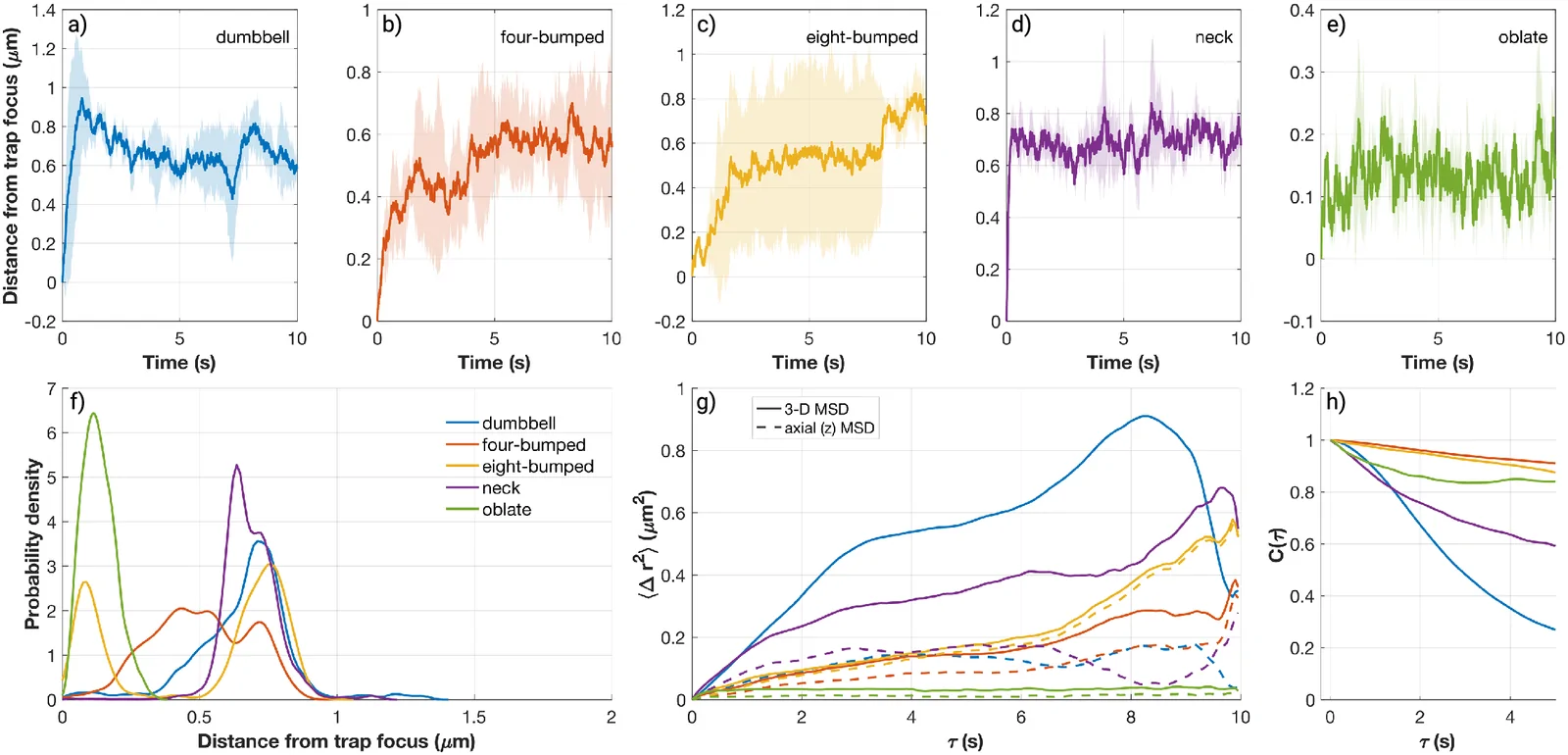
**Dynamic confinement of deformed red blood cell morphologies under a single-beam optical trap.** Top row (a–e): time evolution of the center–focus distance $r=\sqrt{x^{2}+y^{2}+z^{2}}$ (full 3-D Euclidean distance). Solid lines: ensemble mean over three runs with different random initial orientations; shading: one standard deviation. Bottom row (f–h): (f) radial probability density of the same 3-D distance *r*; (g) mean-squared displacement versus lag time *τ*: solid lines = full 3-D center MSD; dashed lines = axial (*z*) MSD; (h) orientation autocorrelation $C(\tau )=\langle \hat{z}(t)\cdot \hat{z}(t+\tau )\rangle$ , where $\hat{z}$ is the unit vector along the cell body *z*-axis in the laboratory frame (orientational memory of the preferred pose, not rotation about a fixed lab axis). All morphologies remain dynamically confined; confinement strength depends on geometry.

The statistical characterization of the confined motion further clarifies these differences. The radial probability density functions (Fig. 8(f)) are sharply peaked near the equilibrium trapping region for all morphologies, confirming stable confinement within a limited spatial region. However, the widths of the distributions vary systematically with geometry. In particular, the oblate cell exhibits the narrowest distribution, consistent with its comparatively large translational stiffness along the *x*-axis (Table 2). In contrast, the four-bumped cell, which displays smaller stiffness values, shows a broader spatial distribution. These differences reflect the combined effects of effective trap stiffness and geometric anisotropy.

The mean-squared displacement (MSD) curves in Fig. 8(g) show the usual crossover from short-time diffusion to longer-time confinement in an optical trap [6,39]. Solid lines give the full 3-D motion of the cell center; dashed lines give only the motion along the beam axis *z*. When a dashed curve lies well below the solid one, most of the MSD is lateral; when the two nearly coincide, the MSD is mainly axial. The oblate cell reaches the lowest long-time MSD, consistent with its large lateral stiffness (Table 2). Other morphologies do not follow a simple one-to-one ranking with lateral *k*: for example, the neck cell has a large translational stiffness but still a comparatively high 3-D MSD, dominated by lateral rather than axial motion. The dashed curves also make the weak optical axial spring explicit (Sec. 4.2): axial MSD often remains large, so practical axial localization relies on the balance between optical force *F_z_* and the buoyancy-corrected cell weight.

Rotational dynamics also vary with cell geometry. The orientation autocorrelation functions (Fig. 8(h)), $C(\tau )=\langle \hat{z}(t)\cdot \hat{z}(t+\tau )\rangle$ with $\hat{z}$ the cell body *z*-axis in the laboratory frame, show that some morphologies lose orientational memory more rapidly than others, consistent with rotational Brownian relaxation in anisotropic trapping potentials [6]. The dumbbell and neck geometries exhibit a faster decay of C(τ) , consistent with their relatively larger rotational stiffness values (see Table 3) and a stronger restoring tendency toward the equilibrium orientation. In contrast, the four-bumped cell displays a slower decay, reflecting its significantly smaller rotational stiffness. The eight-bumped morphology, which has the smallest rotational stiffness in Table 3, shows only weak orientational confinement. The oblate cell exhibits intermediate behavior. Overall, the decay rates of the autocorrelation functions follow the ordering of rotational stiffness obtained from the static torque analysis.

## 6. Conclusions

In this work, we developed a computational framework to investigate how deformed red blood cell (RBC) morphologies affect optical trapping under a single focused beam. While optical trapping of healthy biconcave RBC shapes has been extensively studied [10–12], several of the deformed morphologies considered here have received little or no previous attention in this context. The present study therefore provides a morphology-resolved analysis of optical forces, torques, trap-stiffness anisotropy, and Brownian confinement for a range of physiologically and pathologically relevant RBC geometries.

In the static analysis, each morphology was displaced and rotated about the principal axes, and the resulting optical forces and torques were computed. Near the equilibrium trapping position, these responses exhibited an approximately linear restoring behaviour, allowing translational and rotational trap stiffness values to be extracted from the corresponding slopes. Across all five morphologies we find restoring lateral (transverse) forces and, where symmetry permits, restoring tilt torques. Axial optical forces *F_z_* remain weak near the trapping region and do not provide a clear restoring well about *z* = 0. In a typical upright experiment, practical axial equilibrium is therefore expected from the balance between the optical force *F_z_* and the buoyancy-corrected cell weight ( Supplement 1, Table S1). The Brownian-dynamics simulations (Fig. 8) include optical forces and torques only (no gravitational term is added as, for the deformed morphologies, the buoyancy-corrected weights are very small ( $F_{b}∼10^{-3}–10^{-2}\;\text{pN}$ ; Supplement 1, Table S1), often comparable to characteristic thermal force scales over micrometre displacements) and show bounded center–trap fluctuations over the simulated window for all morphologies, with lateral and tilt confinement as the operative optical trapping criteria. However, the magnitude of the restoring forces and torques varies significantly with geometry. For example, the oblate cell exhibits enhanced translational confinement along specific axes, whereas the eight-bumped morphology generates only weak restoring torques $\left(\kappa ∼10^{-2}\;\text{pN}\cdot \mu m/\text{rad}\right)$ due to its near-spherical symmetry. These findings demonstrate that trapping strength is controlled not only by cell size, but also by geometric anisotropy, symmetry, and local morphological features.

The subsequent Brownian dynamics simulations extend the static analysis by directly testing whether the predicted restoring forces and torques are sufficient to maintain confinement under thermal fluctuations. These simulations confirmed that all considered morphologies remain stably confined over the simulated timescales. The radial probability distributions, mean-squared displacement curves, and orientation autocorrelation functions consistently reflected the stiffness trends obtained from the static analysis. Morphologies exhibiting larger restoring forces and torques displayed reduced fluctuation amplitudes and faster rotational relaxation, demonstrating quantitative agreement between static and dynamic characterisations. This dynamic analysis is a key contribution of the present work, as it links the morphology-dependent stiffnesses to observable confinement statistics and orientational relaxation.

Overall, this study shows that even strongly deformed RBC morphologies can be effectively trapped using a single focused optical beam, although the degree of translational and rotational confinement depends sensitively on cell geometry. The main novelty of the work is twofold: first, it extends optical trapping analysis to deformed RBC geometries that have not been systematically explored before; second, it combines static force and torque calculations with Brownian dynamics to establish a quantitative link between RBC morphology, trap-stiffness anisotropy, and thermal confinement.

Several limitations of the present study should also be noted. The computational framework was validated against the known optical trapping behaviour of a healthy biconcave RBC; however, direct comparison with experimental measurements on the deformed morphologies considered here is not yet available, and systematic optical-trapping studies of such deformed RBC shapes remain comparatively scarce in the literature. The present results may therefore motivate targeted experimental investigations. More generally, recent studies on the optical manipulation of complex and asymmetric micro-objects have shown that optical forces can couple with thermal effects, hydrodynamic fluxes, particle orientation and near-wall interactions, leading to rich dynamical behaviours beyond simple trapping by conservative optical forces [29,40,41]. In the present work, these effects are not explicitly included: hydrodynamic resistance enters the Brownian dynamics through morphology-dependent bulk diffusion tensors, but the optical force calculation does not account for flow-induced perturbations, optically induced fluid motion, near-wall hydrodynamic corrections, or membrane deformation under combined hydrodynamic and optical stresses. Future extensions could couple the present optical-force framework with fluid flow and deformable-membrane models, enabling a more realistic description of the interplay between cell morphology, mechanical response, and trapping dynamics in confined microfluidic environments [4,22].

## Supplemental information

Supplement 1 ESI 1 https://doi.org/10.6084/m9.figshare.33972511

## Data Availability

The custom analysis code used to generate the results presented in this study is publicly available at [42]. Additional simulation data underlying the figures may be obtained from the corresponding author upon reasonable request.

## Supplemental document

See Supplement 1 for supporting content.
