# Supplementary material for: How morphology affects optical trapping of red blood cells in health and disease
Source: Biomed Opt Express. 2026 Sep 30;17(10):5355–72. doi: 10.1364/BOE.609102 (PMC13671685; doi:10.1364/BOE.609102)
Supplement: Supplementary file 1 [file boe-17-10-5355-s001.pdf]

Supplemental Document

Biomedical Optics EXPRESS

How morphology affects optical trapping of red blood cells in health and disease: supplement

EMIR ERDEM,[1] GÖKBERK KABACAOĞLU,[2,*] GIOVANNI VOLPE,[3] AGNESE CALLEGARI,[3] AND LUCA BIANCOFIORE[1,4]

[1]*Department of Mechanical Engineering, Bilkent University, Ankara, Turkey*
[2]*Department of Computer Science, Durham University, Durham, United Kingdom*
[3]*Department of Physics, University of Gothenburg, Gothenburg, Sweden*
[4]*Department of Industrial and Information Engineering and Economics, University of L'Aquila, L'Aquila, Italy*
[*]*gokberk.kabacaoglu@durham.ac.uk*

This supplement published with Optica Publishing Group on 30 September 2026 by The Authors under the terms of the Creative Commons Attribution 4.0 License in the format provided by the authors and unedited. Further distribution of this work must maintain attribution to the author(s) and the published article's title, journal citation, and DOI.

Supplement DOI: https://doi.org/10.6084/m9.figshare.33972511

Parent Article DOI: https://doi.org/10.1364/BOE.609102

Title of primary manuscript:

How Morphology Affects Optical Trapping of Red Blood Cells in Health and Disease

supplemental document

June 15, 2026

Contents

- Section S1: Parametric representation of RBCs
- Section S2: Tuning of optical parameters
- Section S3: Static Analysis
- Section S4: Dynamic Analysis
- Section S5: Numerical Algorithm

1 Parametric representation of RBCs

1.1 Healthy RBC representation

The reference geometry of a healthy red blood cell (RBC) is described using the classical Evans–Fung model [1], which captures the characteristic biconcave shape of the cell. In Cartesian coordinates, the surface is defined implicitly as

$$z^2 = \left(1 - \frac{x^2+y^2}{r^2}\right)\left(C_0 + C_1\frac{x^2+y^2}{r^2} + C_2\frac{(x^2+y^2)^2}{r^4}\right), \tag{S1}$$

where r denotes the cell radius, and C_0, C_1, and C_2 are shape coefficients that determine the thickness profile and degree of concavity.

Following Ref. [2], these coefficients are expressed in terms of physically meaningful geometric parameters, namely the minimum thickness τ_{min}, the maximum thickness τ_{max}, and the radial position d at which the maximum thickness occurs:

$$\begin{aligned} C_0 &= \frac{\tau_{min}}{2r}, \\ C_1 &= \frac{4r^2}{2d^2}\left[-4C_0 + \frac{\tau_{max}(5d^2-16r^2)}{(4r^2-d^2)^{3/2}}\right], \\ C_2 &= \frac{16r^2}{2d^4}\left[2C_0 + \frac{\tau_{max}(16r^2-3d^2)}{\operatorname{sgn}(5d^2-16r^2)(16r^2-d^2)^{3/2}}\right]. \end{aligned} \tag{S2}$$

This parametrization provides direct control over the overall size and thickness distribution of the RBC. The parameter values used for the reference healthy RBC in this work are:

$$r = 3.91\,\mu\text{m}, \quad \tau_{min} = 0.81\,\mu\text{m}, \quad \tau_{max} = 2.52\,\mu\text{m}, \quad d = 2.76\,\mu\text{m}.$$

These values are consistent with experimentally reported RBC dimensions [3] and ensure a realistic baseline geometry for the simulations.

1.2 Spherical harmonic functions

The deformed cell morphologies are partly defined in terms of spherical harmonics. We use the standard complex spherical harmonics

$$Y_\ell^m(\theta,\phi) = (-1)^m \sqrt{\frac{2\ell+1}{4\pi}\frac{(\ell-m)!}{(\ell+m)!}}\, P_\ell^m(\cos\theta)\, e^{im\phi}, \tag{S3}$$

where P_ℓ^m denotes the associated Legendre polynomial, ℓ is the degree, and m is the order. For the present morphologies, the following harmonics are used explicitly.

- For the dumbbell shape,

$$Y_2^0(\theta,\phi) = \sqrt{\frac{5}{16\pi}}\,\left(3\cos^2\theta - 1\right), \tag{S4}$$

  which is purely real, so that

$$\text{Re}\{Y_2^0(\theta,\phi)\} = \sqrt{\frac{5}{16\pi}}\,\left(3\cos^2\theta - 1\right). \tag{S5}$$

- For the four-bumped shape,

$$Y_3^2(\theta,\phi) = \sqrt{\frac{105}{32\pi}}\,\cos\theta\,\sin^2\theta\, e^{2i\phi}, \tag{S6}$$

  and therefore

$$\text{Re}\{Y_3^2(\theta,\phi)\} = \sqrt{\frac{105}{32\pi}}\,\cos\theta\,\sin^2\theta\,\cos(2\phi). \tag{S7}$$

Substituting these expressions into the radial functions used in the main text gives

$$\rho_{\text{dumbbell}}(\theta,\phi) = 1 + \sqrt{\frac{5}{16\pi}}\,\left(3\cos^2\theta - 1\right), \tag{S8}$$

and

$$\rho_{\text{four-bump}}(\theta,\phi) = 1 + 0.2\,\exp\left[-3\sqrt{\frac{105}{32\pi}}\,\cos\theta\,\sin^2\theta\,\cos(2\phi)\right]. \tag{S9}$$

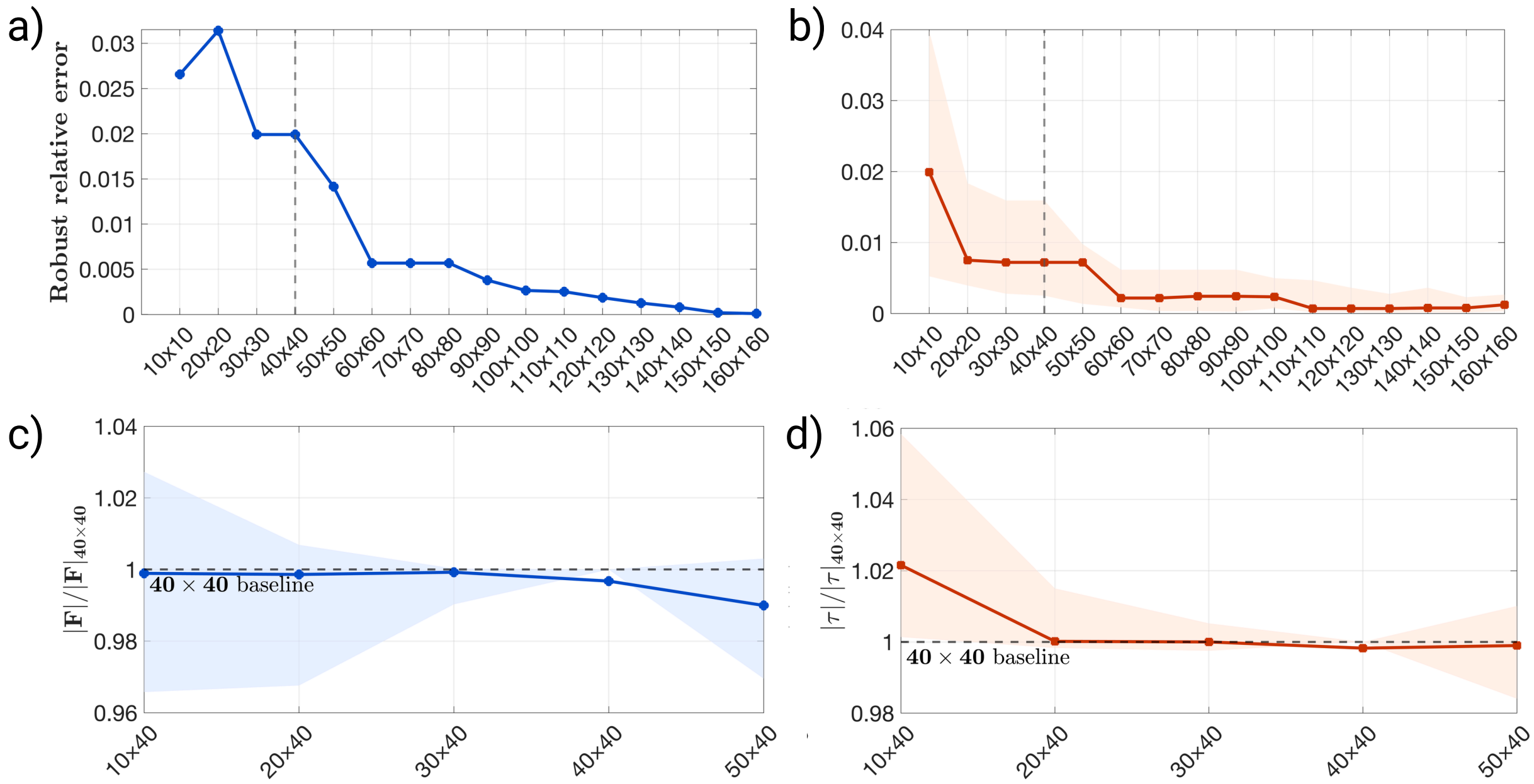

Fig. S1. **Ray-set convergence and fine-resolution sensitivity analysis.** (a) Robust relative error of total optical force and (b) torque magnitudes with respect to the finest resolution (160×160), aggregated across five cell geometries (median with interquartile range). Both quantities converge rapidly, with errors below a few percent beyond approximately 40×40. (c)–(d) Fine aspect-ratio sweep for non-square ray sets (10×40–50×40), normalized by the 40×40 baseline. The small variation, within $\sim 2\%$, confirms that reducing the azimuthal ray count does not materially affect accuracy, supporting the use of a 20×40 ray set as an efficient and reliable configuration for subsequent simulations.

2 Tuning of optical parameters

The optical forces and torques were computed using the OTGO framework [4] within the ray-optics approximation [5]. Three numerical parameters were tuned to ensure a reliable compromise between accuracy and computational cost: the ray-set discretisation, the surface tessellation density, and the maximum number of scattering events retained in the ray-tracing calculation.

To assess convergence with respect to ray discretisation, the number of azimuthal and radial divisions was first varied simultaneously from 10×10 to 160×160, in increments of 10 divisions in each direction. The resulting ray sets were applied to representative cell geometries, and the corresponding force and torque components were recorded. As shown in Fig. S1a,b, the relative errors in both force and torque magnitudes decrease rapidly with increasing ray resolution and fall below a few percent beyond approximately 40×40. Additional asymmetric ray sets were then tested to reduce computational cost while maintaining accuracy. The fine-resolution sweep shown in Fig. S1c,d demonstrates that reducing the azimuthal resolution while keeping 40 radial divisions produces only small variations, within approximately 2%, relative to the 40×40 baseline. Based on these tests, a 20×40 azimuthal–radial ray discretisation was selected as an efficient compromise between accuracy and computational cost.

The cell surface was discretised using a Gauss–Legendre–uniform grid in the parametric coordinates (u, v), with $u \in [0, \pi]$ and $v \in [0, 2\pi)$. The discretisation parameter p controls the surface sampling: v is divided into $2p$ uniform intervals, while u is sampled using $p + 1$ Gauss–Legendre nodes. This yields

$$N = (p + 1)(2p) \approx 2p^2$$

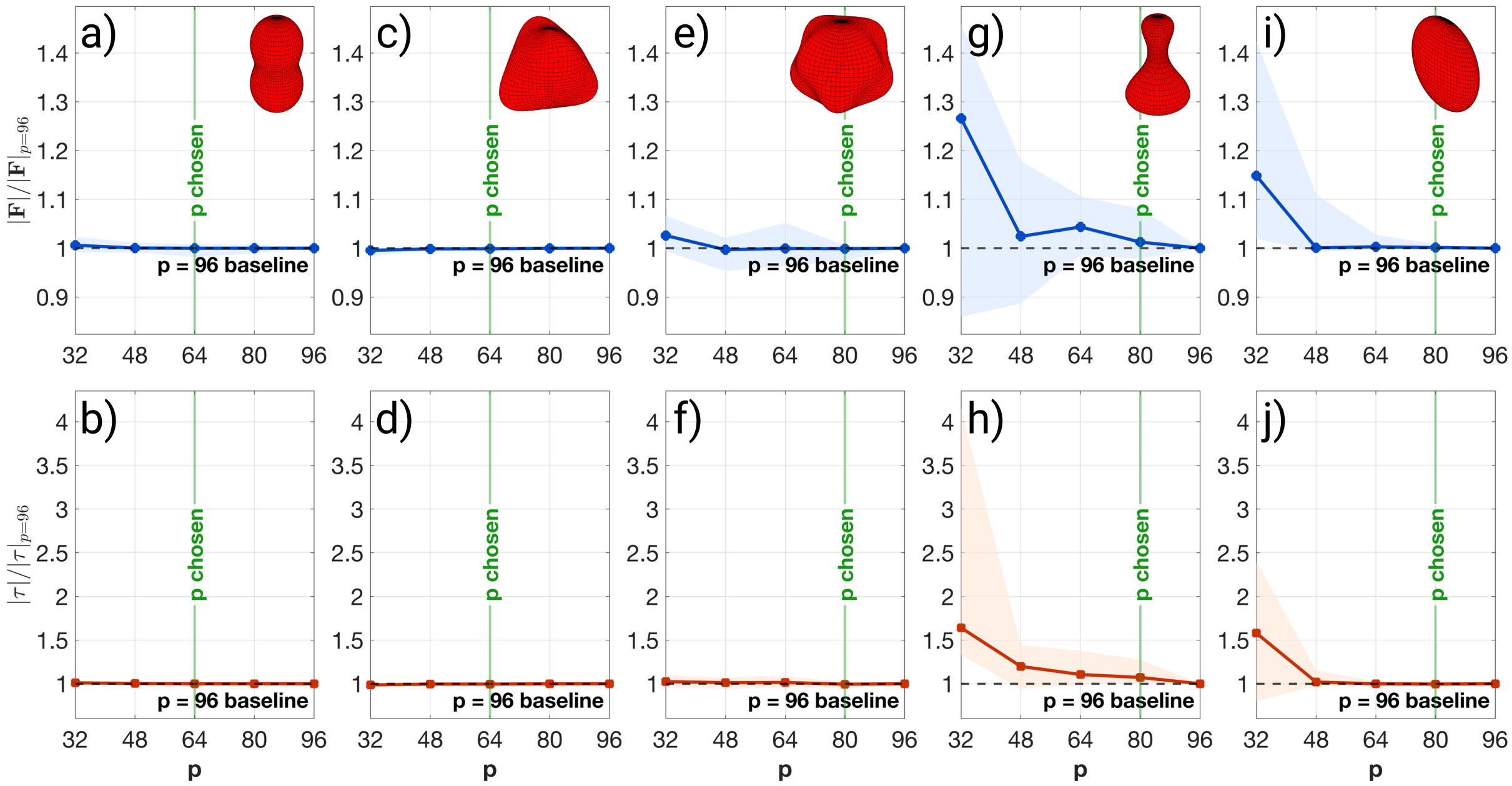

Fig. S2. **Sensitivity of optical forces and torques to the surface tessellation density p.** For each cell geometry, the total force and torque were evaluated along a short x-displacement sweep and summarised as the median, with interquartile range shown by the shaded region, of the normalized magnitudes relative to the finest mesh ($p = 96$). Panels (a)–(e) show $|\mathbf{F}|/|\mathbf{F}|_{p=96}$, and panels (f)–(j) show $|\boldsymbol{\tau}|/|\boldsymbol{\tau}|_{p=96}$. The dashed horizontal line marks the $p = 96$ baseline, and the vertical green line indicates the value used in production for each cell: dumbbell $p = 64$, four-bumped $p = 64$, eight-bumped $p = 80$, neck $p = 80$, and oblate $p = 80$. Across all geometries, both $|\mathbf{F}|$ and $|\boldsymbol{\tau}|$ vary only weakly beyond these choices, confirming that the selected p values provide sufficient tessellation density while maintaining computational efficiency.

surface points. Thus, p sets the tessellation density: larger values of p refine the mesh and improve the accuracy of geometric quantities, such as normals and curvatures, and of derived observables, such as optical forces and torques, at the cost of greater computational effort.

To evaluate sensitivity to surface discretisation, p was varied from 32 to 96 in steps of 16, corresponding to $N = 2112$, 4704, 8320, 12960, and 18624 surface points. For each value of p, the cells were displaced along the x-axis in small increments, and the resulting optical forces and torques were recorded. Figure S2 shows that the normalized force and torque magnitudes vary only weakly once the tessellation density is sufficiently refined. The required value of p is nevertheless morphology-dependent, with sharper features and narrow necks requiring finer sampling. The production value of p was therefore chosen as the smallest value for which further refinement produced negligible changes in the computed forces and torques. The selected values were $p = 64$ for the dumbbell and four-bumped cells, and $p = 80$ for the eight-bumped, neck, and oblate cells.

Finally, the number of scattering events retained in the ray-tracing calculation was tuned. Tracing reflected and refracted rays within the cell becomes unnecessary beyond a certain number of events, because the power carried by successive rays decreases progressively and their contribution to the net optical forces and torques becomes negligible. To quantify this effect, up to 50 scattering events were traced for a fixed configuration of each of the five cell morphologies, and the total power associated with the final ray set was recorded. As shown in Fig. S3, the residual power decreases rapidly and becomes negligible after approximately 10 scattering events for all geometries. This value was therefore adopted as the cutoff for subsequent force and torque calculations.

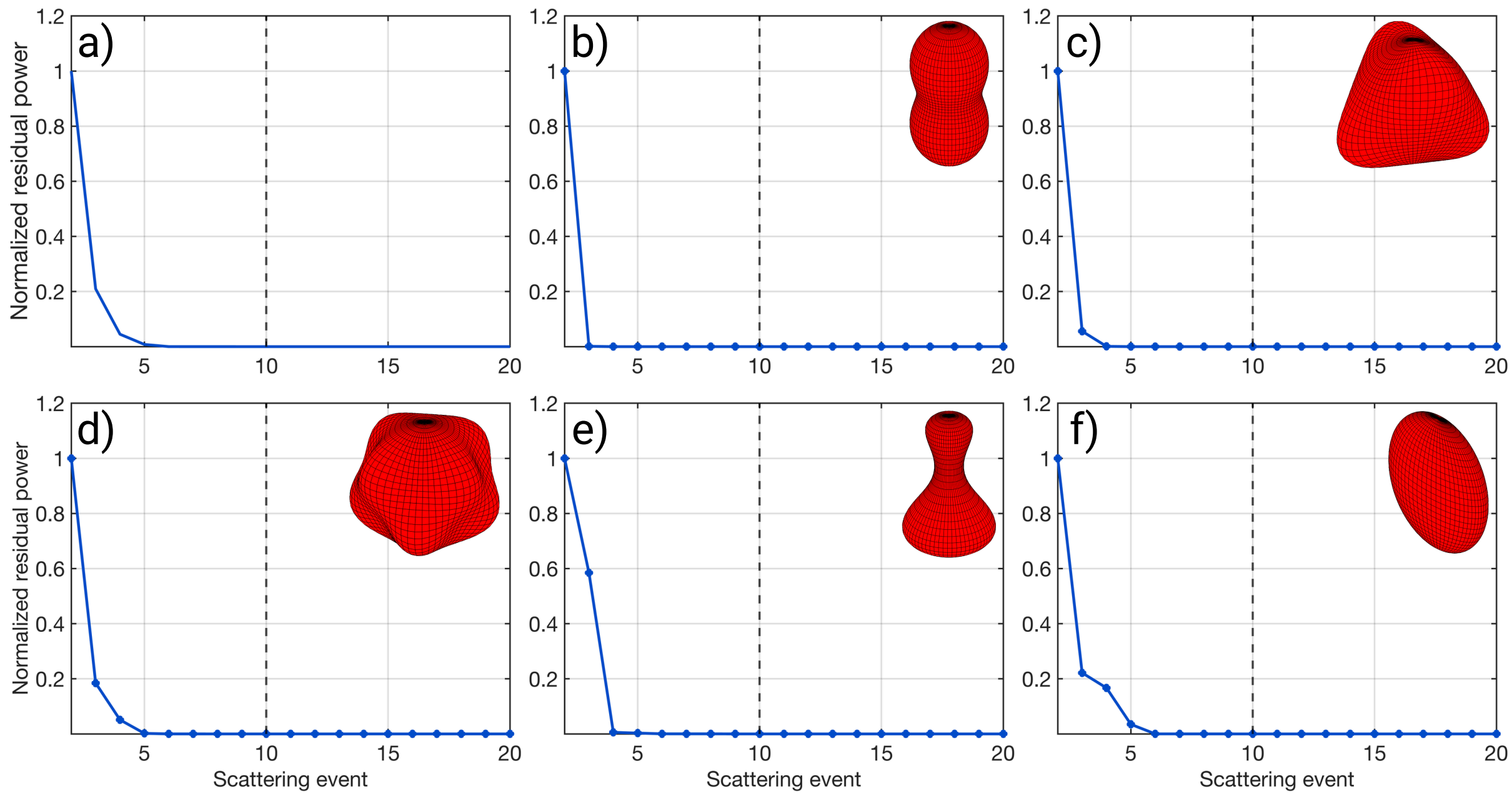

Fig. S3. **Normalized residual power in the final ray set as a function of the number of scattering events for different cell geometries.** (a) Aggregate behaviour across all five cell types; (b)–(f) individual results for the dumbbell, four-bumped, eight-bumped, neck, and oblate cells, respectively. The residual power, normalized by its value after the second scattering event, decays rapidly and becomes negligible after approximately ten events for all shapes. This confirms that limiting ray tracing to ten scattering events provides a sufficient cutoff for accurate evaluation of optical forces and torques while minimizing computational cost. Only the first twenty scattering events are shown for clarity.

3 Static Analysis

3.1 Axial force response during z-axis displacement

The axial force response was also examined by displacing each morphology along the beam propagation direction. As shown in Fig. S4, the axial force F_z generally reaches its largest values when the cells are positioned below the focal plane, where the interaction with the focused beam is strongest. As the cells are displaced towards and above the equilibrium trapping region, the axial force decreases substantially and remains small in the neighbourhood of the trap centre. Although the curves do not exhibit a clear linear restoring regime along the z-axis, the small magnitude of F_z near the trapping position indicates that radiation-pressure or scattering contributions do not destabilise the lateral optical confinement. This confirms that stable trapping is primarily governed by the transverse restoring forces and torques discussed in the main static analysis.

3.2 Geometric anisotropy measures

To quantify the geometric anisotropy of the modelled cell shapes, each surface was approximated by an equivalent ellipsoid derived from the spatial distribution of its surface points. Let $\{\mathbf{x}_i\}_{i=1}^{N}$ denote the Cartesian coordinates of the discretised surface. The centroid of the shape is first computed as

$$\bar{\mathbf{x}} = \frac{1}{N}\sum_{i=1}^{N}\mathbf{x}_i. \tag{S10}$$

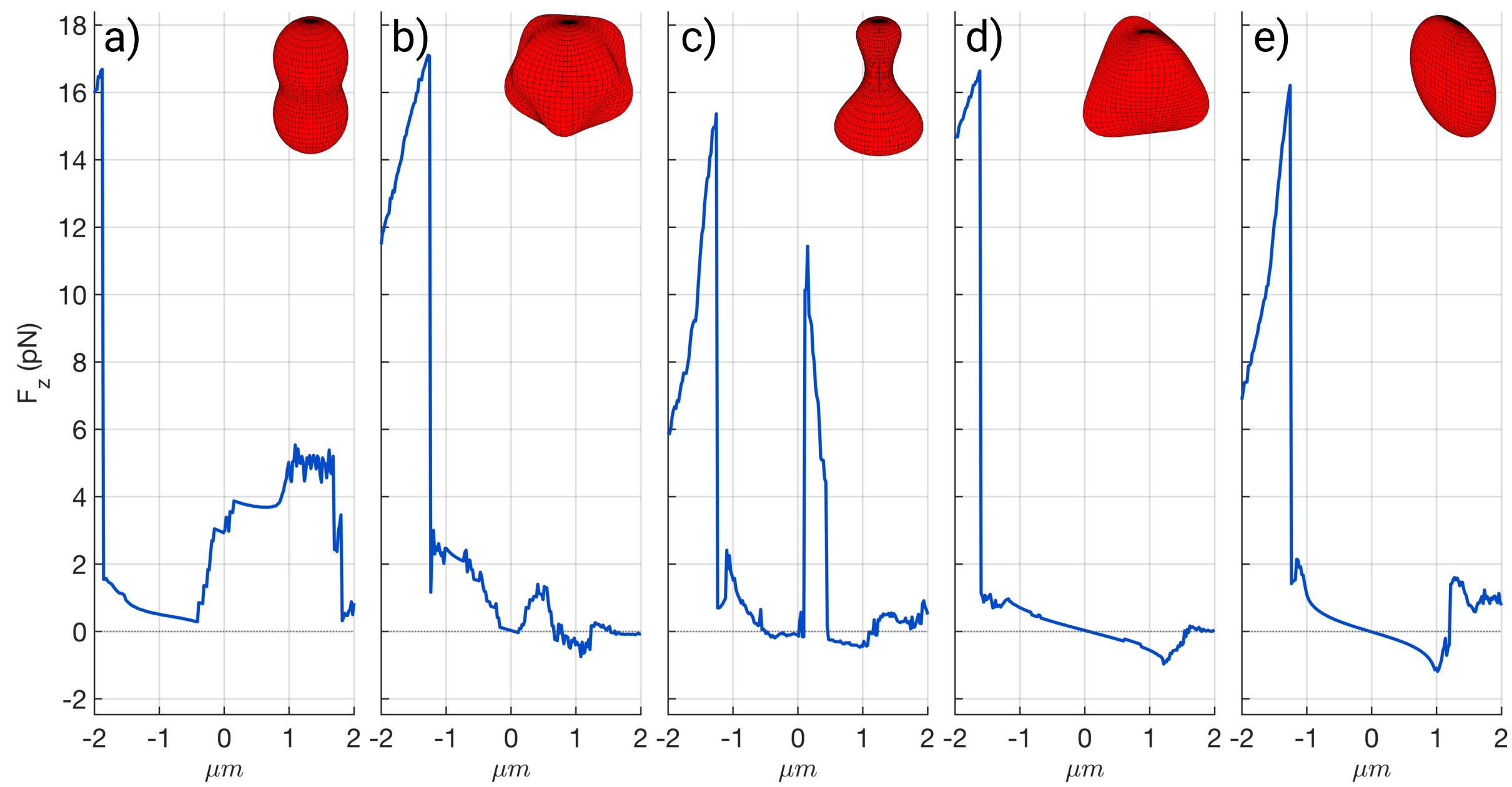

Fig. S4. **Axial force response for each cell geometry during z-axis displacement.** The plotted quantity is the optical force along the beam axis, F_z, expressed in piconewtons. Each panel corresponds to a different morphology: (a) dumbbell, (b) eight-bumped, (c) neck, (d) four-bumped, and (e) oblate cells. Forces are largest when the cells are positioned below the focal plane and decrease near the equilibrium trapping region, indicating that axial scattering forces remain too weak to destabilise the optical trap.

The covariance matrix of the surface points is then defined as

$$\mathbf{C} = \frac{1}{N}\sum_{i=1}^{N}(\mathbf{x}_i - \bar{\mathbf{x}})(\mathbf{x}_i - \bar{\mathbf{x}})^{\mathrm{T}}. \tag{S11}$$

The eigenvalues $\lambda_1 \geq \lambda_2 \geq \lambda_3$ of $\mathbf{C}$ correspond to the principal spatial variances of the shape. The semi-axes of the equivalent ellipsoid are therefore defined as

$$a = \sqrt{\lambda_1}, \quad b = \sqrt{\lambda_2}, \quad c = \sqrt{\lambda_3}. \tag{S12}$$

The aspect ratio used in the main text is then given by

$$\frac{a}{c}, \tag{S13}$$

which provides a simple scalar measure of global directional anisotropy. In addition to the aspect ratio, we also define the asphericity as

$$\mathcal{A} = \frac{(a-b)^2 + (b-c)^2 + (c-a)^2}{2(a+b+c)^2}, \tag{S14}$$

which quantifies the overall deviation from a spherical geometry. By construction, $\mathcal{A} = 0$ for a perfect sphere and increases with shape anisotropy.

Figure S5 shows the mean lateral trap stiffness as a function of the asphericity $\mathcal{A}$. While the general trend of increasing stiffness with geometric anisotropy is preserved, the correlation is less monotonic than that obtained using the aspect ratio a/c. In particular, morphologies with distinct

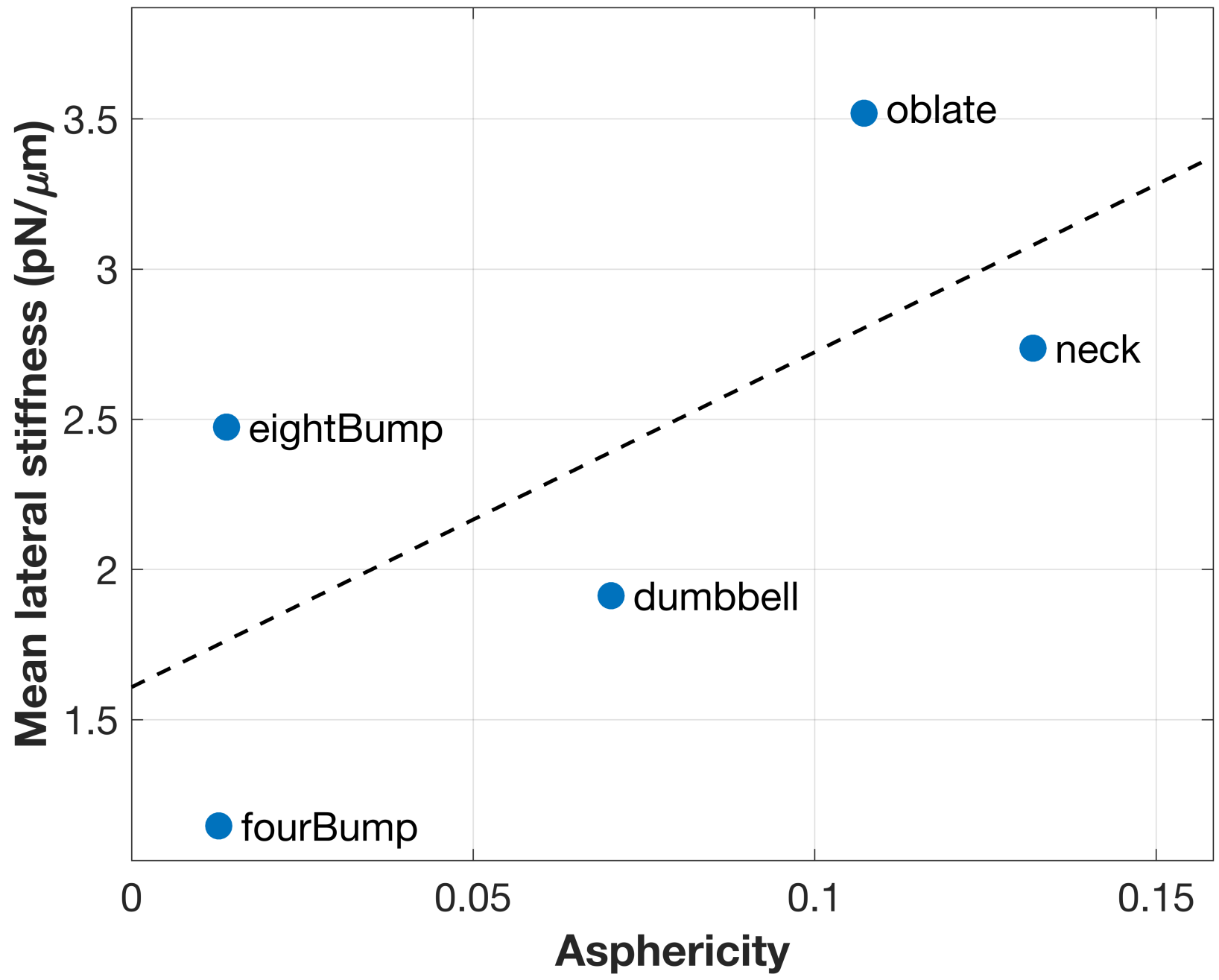

Fig. S5. **Mean lateral trap stiffness as a function of asphericity $\mathcal{A}$ for the different cell morphologies.** While a general increase in stiffness with anisotropy is observed, the correlation is less monotonic than that obtained using the aspect ratio.

geometric features but similar levels of global anisotropy, such as the dumbbell and eight-bumped cells, are not clearly separated by the asphericity measure.

This difference arises because asphericity provides a scalar measure of the overall deviation from sphericity, but does not distinguish between different types of anisotropy, such as elongation, flattening, or local shape modulation. In contrast, the aspect ratio a/c directly captures the strongest directional geometric asymmetry, which can strongly influence optical forces and torques in non-spherical particles [6, 7].

In particular, the eight-bumped morphology exhibits relatively high stiffness despite its low asphericity, reflecting the influence of local surface features and symmetry on the optical response. This highlights the limitation of purely global scalar descriptors for complex cell geometries. Overall, these results indicate that, while asphericity provides a useful general measure of deviation from spherical shape, the aspect ratio offers a more physically relevant and predictive metric for correlating geometry with trap stiffness in the present system.

4 Dynamic Analysis

4.1 Determination of diffusion tensor

The diffusion tensor is a 6×6 matrix consisting of 3×3 sub-blocks accounting for translational and rotational degrees of freedom, as well as their hydrodynamic coupling [8]. The diffusion matrix depends on the cell's size, shape, orientation, and on the temperature and viscosity of the surrounding medium. In the present simulations, the temperature was set to $T = 293\,\mathrm{K}$ and the viscosity of water to $\eta = 0.001\,\mathrm{Pa \cdot s}$.

To determine the diffusion tensors of the different cell geometries, a numerical bead-model approach was employed. In this method, the complex particle geometry is approximated by an assembly of spherical beads that cover its surface. Hydrodynamic interactions among the beads are then used to compute the resistance tensor of the model particle [9].

The software `winHYDRO++` implements this approach efficiently [9, 10]. Given the Cartesian coordinates and radii of the beads, the program evaluates the full 6×6 resistance tensor $\boldsymbol{\Xi}$, encoding translational, rotational, and coupling friction coefficients. The resulting tensors therefore correspond to bulk-fluid hydrodynamics; near-wall corrections, which can modify translational and rotational drag close to surfaces, are not included in the present simulations [11]. The diffusion tensor $\mathbf{D}$ is then obtained via the generalized Einstein relation [8]:

$$\mathbf{D} = k_B T \, \boldsymbol{\Xi}^{-1}, \tag{S15}$$

where k_B is the Boltzmann constant and T the absolute temperature.

4.2 Time-step selection and characteristic timescales

The integration time step Δt in Brownian dynamics simulations must be chosen with respect to the characteristic timescales governing the system. In the overdamped regime relevant to optically trapped cells, the two main timescales are the trap relaxation time,

$$\tau_{\mathrm{OT}} = \frac{\gamma}{k}, \tag{S16}$$

and the momentum relaxation time,

$$\tau_m = \frac{m}{\gamma}, \tag{S17}$$

where γ is the hydrodynamic friction coefficient, k is the trap stiffness, and m is the particle mass [12, 13].

The friction coefficients were obtained from the diffusion tensor $\mathbf{D}$ using the fluctuation–dissipation relation $\gamma = k_B T / D$, evaluated along the principal directions of motion. Translational and rotational relaxation times were then computed for each morphology using the corresponding stiffness values extracted from the static force and torque analyses.

The resulting characteristic timescales are summarised in Table S1. Across all geometries, the smallest trap relaxation time is obtained for the oblate cell along the x-direction, with $\tau_{\min} \approx 2.6 \times 10^{-2}\,\mathrm{s}$. For comparison, using a representative RBC mass of $m \sim 10^{-11}\,\mathrm{kg}$ [8] and the corresponding friction coefficients, the momentum relaxation time is estimated to be $\tau_m \sim 10^{-4}\,\mathrm{s}$. This confirms the overdamped hierarchy $\tau_m \ll \tau_{\mathrm{OT}}$ for all cases.

These estimates establish the practical range for the integration time step. The time step must be sufficiently smaller than the trap relaxation time to resolve the confined Brownian dynamics, while remaining much larger than the momentum relaxation time, which is not explicitly resolved in the overdamped formulation. Thus, the relevant hierarchy is

$$\tau_m \ll \Delta t \lesssim \tau_{\mathrm{OT}}. \tag{S18}$$

Although a strict condition $\Delta t \ll \tau_{\mathrm{OT}}$ is not always required for numerical stability, excessively large time steps lead to inaccurate dynamics. This is confirmed by the time-step sensitivity analysis shown in Fig. 6 of the main text, where stable confinement is observed for $\Delta t \leq 3\,\mathrm{ms}$, while larger values result in artificial drift.

Table S1. Characteristic relaxation times for each morphology, computed from the diffusion tensor and trap stiffness values, together with the corresponding volume and surface area. Translational (τ_t) and rotational (τ_r) relaxation times are reported for the x- and y-directions.

Shape	$\tau_{t,x}$ (s)	$\tau_{t,y}$ (s)	$\tau_{r,x}$ (s)	$\tau_{r,y}$ (s)	$\tau_{\min}$ (s)	V (μm^3)	A (μm^2)
Dumbbell	0.117	0.117	0.379	0.379	0.117	5.236	16.200
Four-bumped	0.204	0.212	3.82	5.81	0.204	8.273	21.164
Eight-bumped	0.077	0.077	–	–	0.077	4.271	13.505
Neck	0.047	0.047	0.076	0.076	0.047	0.922	5.696
Oblate	0.026	0.098	–	0.088	0.026	1.531	7.783

4.3 Time-step update of position and orientation

The translational and rotational dynamics were evolved using an overdamped Brownian dynamics scheme following Refs. [8, 14]. Translational and rotational increments are first computed in the particle frame Σ_p, and then mapped to the laboratory frame Σ_ℓ through the rotation matrix $\mathbf{M}_{p\to\ell}(t) \in \mathbb{R}^{3\times3}$. Its transpose, $\mathbf{M}_{\ell\to p}(t) = \mathbf{M}^{\mathrm{T}}_{p\to\ell}(t)$, maps vectors from the laboratory frame to the particle frame. We define

$$\boldsymbol{q}_p \equiv \begin{bmatrix} \Delta x_p \\ \Delta y_p \\ \Delta z_p \\ \Delta\alpha_p \\ \Delta\beta_p \\ \Delta\gamma_p \end{bmatrix}, \qquad \boldsymbol{f}_p \equiv \begin{bmatrix} \mathbf{F}_p \\ \mathbf{T}_p \end{bmatrix}, \qquad \mathbf{D} \in \mathbb{R}^{6\times6},$$

where $\boldsymbol{q}_p$ contains the translational and rotational increments, $\boldsymbol{f}_p$ contains the optical force and torque, and $\mathbf{D}$ is the full translational–rotational diffusion tensor.

At each time step Δt, the increment in the particle frame is computed as

$$\boldsymbol{q}_p = \frac{\mathbf{D}}{k_B T}\,\boldsymbol{f}_p\,\Delta t + \sqrt{2\Delta t}\,\mathbf{L}\,\boldsymbol{\xi}, \qquad \boldsymbol{\xi} \sim \mathcal{N}(\mathbf{0}, \mathbf{I}_6), \qquad \mathbf{D} = \mathbf{L}\mathbf{L}^{\mathrm{T}}, \tag{S19}$$

where $\mathbf{L}$ is the Cholesky factor of $\mathbf{D}$. The stochastic term therefore satisfies

$$\mathrm{Cov}\Big[\sqrt{2\Delta t}\,\mathbf{L}\boldsymbol{\xi}\Big] = 2\mathbf{D}\Delta t,$$

in accordance with the generalized fluctuation–dissipation relation [8, 14].

The optical force and torque are first computed in the laboratory frame and then expressed in the particle frame as

$$\mathbf{F}_p = \mathbf{M}_{\ell\to p}(t)\,\mathbf{F}_\ell, \qquad \mathbf{T}_p = \mathbf{M}_{\ell\to p}(t)\,\mathbf{T}_\ell, \qquad \mathbf{M}_{\ell\to p}(t) = \mathbf{M}^{\mathrm{T}}_{p\to\ell}(t). \tag{S20}$$

The translational position is then updated in the laboratory frame using the first three components of $\boldsymbol{q}_p$:

$$\mathbf{r}_\ell(t+\Delta t) = \mathbf{r}_\ell(t) + \mathbf{M}_{p\to\ell}(t) \begin{bmatrix} \Delta x_p \\ \Delta y_p \\ \Delta z_p \end{bmatrix}. \tag{S21}$$

The rotational motion is represented by the small angular increments $(\Delta\alpha_p, \Delta\beta_p, \Delta\gamma_p)$. Following the convention used in the numerical implementation, the incremental rotation matrix in the

particle frame is written as

$$R_x(\Delta\gamma_p) = \begin{bmatrix} 1 & 0 & 0 \\ 0 & \cos\Delta\gamma_p & -\sin\Delta\gamma_p \\ 0 & \sin\Delta\gamma_p & \cos\Delta\gamma_p \end{bmatrix}, \tag{S22}$$

$$R_y(\Delta\beta_p) = \begin{bmatrix} \cos\Delta\beta_p & 0 & \sin\Delta\beta_p \\ 0 & 1 & 0 \\ -\sin\Delta\beta_p & 0 & \cos\Delta\beta_p \end{bmatrix}, \tag{S23}$$

$$R_z(\Delta\alpha_p) = \begin{bmatrix} \cos\Delta\alpha_p & -\sin\Delta\alpha_p & 0 \\ \sin\Delta\alpha_p & \cos\Delta\alpha_p & 0 \\ 0 & 0 & 1 \end{bmatrix}, \tag{S24}$$

$$\mathbf{R}_p = R_z(\Delta\alpha_p)\, R_y(\Delta\beta_p)\, R_x(\Delta\gamma_p). \tag{S25}$$

Finally, the orientation matrix in the laboratory frame is updated as

$$\mathbf{M}_{p\to\ell}(t+\Delta t) = \mathbf{M}_{p\to\ell}(t)\,\mathbf{R}_p. \tag{S26}$$

5 Numerical Algorithm

To compute the optical forces and torques acting on generic cell shapes, a new `cell` class and the associated geometric functionals were implemented within the computational toolbox OTGO (Optical Tweezers in the Geometrical Optics Approximation) [4]. This implementation allows arbitrary smooth cell morphologies to be represented parametrically, discretised on a surface mesh, and used directly in the ray-optics calculation of optical forces and torques.

5.1 Construction of cells

Each cell object is described by a centre position

$$\mathbf{c} = [p_x, p_y, p_z],$$

three orthonormal orientation vectors,

$$\mathbf{v}_i = [v_i^x, v_i^y, v_i^z], \qquad i = 1, 2, 3,$$

and a discretisation parameter p, which controls the tessellation density of the surface. The orientation vectors define the rotation matrix that maps quantities between the cell-fixed reference frame and the laboratory frame. In this way, the intrinsic geometry of the cell can be constructed once in a reference configuration and then translated or rotated rigidly during the optical-trapping calculations.

Each cell is additionally identified by a string specifying its geometry, namely *dumbbell*, *four-bumped*, *eight-bumped*, *neck*, or *oblate*. This identifier is passed to a geometry-construction routine that generates the corresponding smooth reference surface in the cell-fixed frame using the parametric representations described in the Mathematical Model section of the main text. The surface is then discretised on a Gauss–Legendre–uniform grid in the angular parameters (u, v), yielding $N = 2p(p+1)$ surface points. At each grid point, the Cartesian coordinates of the surface are stored as

$$\mathbf{X}(u_i, v_j) = \big(x(u_i, v_j),\ y(u_i, v_j),\ z(u_i, v_j)\big),$$

providing direct access to the physical location of the surface mesh in three-dimensional space.

5.2 Geometric attributes and surface access

In addition to the surface coordinates, the cell object stores a set of geometric quantities defined pointwise on the discretised surface. First- and second-order partial derivatives of the surface with respect to the parametric coordinates are evaluated spectrally,

$$\mathbf{X}_u,\ \mathbf{X}_v,\ \mathbf{X}_{uu},\ \mathbf{X}_{uv},\ \mathbf{X}_{vv},$$

allowing the local surface geometry to be characterised with high accuracy. From these derivatives, the unit surface normal vector $\mathbf{n}$ and the surface area element W are computed at every grid point. The mean curvature H and Gaussian curvature K are also evaluated on the same grid, providing a complete local geometric description of the cell surface.

The cell class further defines surface differential operators acting on scalar or vector fields sampled on the grid. In particular, the surface gradient ∇_s, surface divergence $\nabla_s\cdot$, and Laplace–Beltrami operator Δ_s are constructed using the metric coefficients of the parametrisation. These operators are implemented as numerical mappings that take discrete surface fields as input and return the corresponding surface derivatives at the grid points. Although not all of these quantities are required for the optical force calculation itself, they provide a general geometric infrastructure for analysing and manipulating complex cell surfaces.

All geometric quantities are defined intrinsically in the cell-fixed reference frame. Rigid-body translations and rotations are applied separately through the centre position $\mathbf{c}$ and orientation vectors $\mathbf{v}_i$, ensuring that the intrinsic surface geometry remains unchanged while the cell moves and reorients in space. This organisation allows the numerical algorithm to access, at any time, the Cartesian surface points, surface normals, and local geometric properties required for evaluating ray–surface intersections and the associated optical forces and torques.

5.3 Ray–surface intersection algorithm

To evaluate optical interactions, the intersection points between incident rays and the cell surface are determined numerically. Each ray is represented as a straight line

$$\mathbf{r}(t) = \mathbf{p}_1 + t\,\mathbf{d}, \tag{S27}$$

where $\mathbf{p}_1$ is a point on the ray, $\mathbf{d}$ is the ray direction, and t is a scalar parameter. For each ray, the algorithm identifies either the first intersection point, corresponding to entry into the cell, or the second intersection point, corresponding to exit from the cell.

All intersection calculations are performed in the cell-fixed reference frame. The ray is first translated by the cell centre and rotated using the orientation matrix, so that it interacts directly with the reference surface geometry. Once the intersection has been identified, the corresponding point is mapped back to the laboratory frame. This avoids repeatedly reconstructing the surface in the laboratory frame and ensures that the same intrinsic surface representation is used throughout the calculation.

An initial interval $[t_{\min}, t_{\max}]$ along the ray is estimated using a bounding box enclosing the discretised surface. Within this interval, the ray is sampled at a finite set of parameter values $\{t_k\}$. At each sample point, the minimum Euclidean distance between the ray point and all surface grid points $\{\mathbf{X}_j\}$ is computed,

$$d_k = \min_j \|\mathbf{r}(t_k) - \mathbf{X}_j\|\,. \tag{S28}$$

Local minima of the resulting distance profile identify candidate regions in which the ray approaches the surface.

The neighbourhood of the first detected local minimum is then refined iteratively by re-sampling a smaller interval along the ray and recomputing the distance profile. After refinement, the parameter value t^* corresponding to the minimum distance is accepted as an intersection if the distance falls below a prescribed tolerance. Otherwise, the ray is considered non-intersecting and the intersection point is marked as undefined.

The tolerance is expressed in dimensionless units relative to the surface discretisation scale. It was set to 0.25 for the dumbbell, four-bumped, neck, and oblate geometries, and to 0.5 for the eight-bumped cell. The larger tolerance for the eight-bumped morphology accounts for its greater surface complexity and stronger local curvature variations. In general, the tolerance is geometry-dependent and was chosen to balance intersection accuracy with numerical robustness. This distance-based procedure provides a robust method for locating ray entry and exit points on arbitrarily shaped cell surfaces without requiring an explicit analytic solution of the ray–surface intersection problem.

5.4 Surface-normal and tangent-plane construction

Several steps of the ray-optics calculation, including reflection, refraction, and the determination of local surface orientation, require the surface normal and tangent plane at the ray–surface intersection point. These quantities are obtained numerically from the discretised surface representation. Given a point $\mathbf{p}$ lying on, or very close to, the cell surface, the local surface normal is estimated by interpolating the normals of nearby surface grid points.

Specifically, for each query point, the Euclidean distances to all discretised surface points $\{\mathbf{X}_j\}$ are computed, and a small set of nearest neighbours is selected. The corresponding surface normals $\{\mathbf{n}_j\}$ are then combined using inverse-distance weighting,

$$\mathbf{n}(\mathbf{p}) = \frac{\sum_j w_j \, \mathbf{n}_j}{\left\| \sum_j w_j \, \mathbf{n}_j \right\|}, \qquad w_j = \frac{1}{\|\mathbf{p} - \mathbf{X}_j\|}, \tag{S29}$$

yielding a smoothly interpolated unit normal at the query point. All calculations are first carried out in the cell-fixed reference frame and are subsequently mapped back to the laboratory frame.

The tangent plane at $\mathbf{p}$ is then constructed as the plane passing through $\mathbf{p}$ and orthogonal to the interpolated surface normal. This provides a consistent local linear approximation of the surface geometry at the intersection point and is used in the subsequent optical ray–surface interaction calculations.

References

[1] E. Evans and Y.-C. Fung, "Improved measurements of the erythrocyte geometry," Microvascular Research **4**, 335–347 (1972).

[2] R. Tognato, "Theoretical investigation of erythrocytes optical trapping in ray optics approximation," Ph.D. thesis, University College London (2022).

[3] M. Diez-Silva, M. Dao, J. Han, C.-T. Lim, and S. Suresh, "Shape and biomechanical characteristics of human red blood cells in health and disease," MRS Bulletin **35**, 382–388 (2010).

[4] A. Callegari, M. Mijalkov, A. B. Gököz, and G. Volpe, "Computational toolbox for optical tweezers in geometrical optics," Journal of the Optical Society of America B **32**, B11–B19 (2015).

[5] A. Ashkin, "Forces of a single-beam gradient laser trap on a dielectric sphere in the ray optics regime," Biophysical Journal **61**, 569–582 (1992).

[6] T. A. Nieminen, V. L. Y. Loke, A. B. Stilgoe, G. Knöner, A. M. Brańczyk, N. R. Heckenberg, and H. Rubinsztein-Dunlop, "Optical tweezers computational toolbox," Journal of Optics A: Pure and Applied Optics **9**, S196 (2007).

[7] S. Simpson and S. Hanna, "Optical trapping of spheroidal particles in gaussian beams," Journal of the Optical Society of America A **24**, 430–443 (2007).

[8] R. Tognato, D. Bronte Ciriza, O. M. Maragò, and P. H. Jones, "Modelling red blood cell optical trapping by machine learning improved geometrical optics calculations," Biomedical Optics Express **14**, 3748–3762 (2023).

[9] J. García de la Torre, S. Navarro, M. C. López Martínez, F. G. Díaz, and J. J. López Cascales, "HYDRO: a computer program for the prediction of hydrodynamic properties of macromolecules," Biophysical Journal **67**, 530–531 (1994).

[10] A. Ortega, D. Amorós, and J. García de la Torre, "Improved calculation of rotational diffusion and intrinsic viscosity of bead models for macromolecules and nanoparticles," The Journal of Chemical Physics **127**, 085102 (2007).

[11] E. Schäffer, S. F. Nørrelykke, and J. Howard, "Surface forces and drag coefficients of microspheres near a plane surface measured with optical tweezers," Langmuir **23**, 3654–3665 (2007).

[12] G. Volpe and G. Volpe, "Simulation of a Brownian particle in an optical trap," American Journal of Physics **81**, 224–230 (2013).

[13] P. Jones, O. Maragò, and G. Volpe, *Optical Tweezers: Principles and Applications* (Cambridge University Press, 2015).

[14] M. X. Fernandes and J. García de la Torre, "Brownian dynamics simulation of rigid particles of arbitrary shape in external fields," Biophysical Journal **83**, 3039–3048 (2002).
